# Selection shapes turnover and magnitude of sex-biased expression in Drosophila gonads

**DOI:** 10.1186/s12862-019-1377-4

**Published:** 2019-02-20

**Authors:** Carrie A. Whittle, Cassandra G. Extavour

**Affiliations:** 1 Department of Organismic and Evolutionary Biology, Harvard University, 16 Divinity Avenue, Cambridge, MA 02138 USA; 2 Department of Molecular and Cellular Biology, Harvard University, 16 Divinity Avenue, Cambridge, MA 02138 USA

**Keywords:** Testes, Ovaries, Sex-biased expression, Turnover, Fold bias, Selection, Pleiotropy, Expression evolution, Protein divergence, Drosophila

## Abstract

**Background:**

Sex-biased gene expression is thought to drive the phenotypic differences in males and females in metazoans. Drosophila has served as a primary model for studying male-female differences in gene expression, and its effects on protein sequence divergence. However, the forces shaping evolution of sex-biased expression remain largely unresolved, including the roles of selection and pleiotropy. Research on sex organs in Drosophila, employing original approaches and multiple-species contrasts, provides a means to gain insights into factors shaping the turnover and magnitude (fold-bias) of sex-biased expression.

**Results:**

Here, using recent RNA-seq data, we studied sex-biased gonadal expression in 10,740 protein coding sequences in four species of Drosophila, *D. melanogaster*, *D. simulans*, *D. yakuba* and *D. ananassae* (5 to 44 My divergence). Using an approach wherein we identified genes with lineage-specific transitions (LSTs) in sex-biased status (amongst testis-biased, ovary-biased and unbiased; thus, six transition types) standardized to the number of genes with the ancestral state (S-LSTs), and those with clade-wide expression bias status, we reveal several key findings. First, the six categorical types of S-LSTs in sex-bias showed disparate rates of turnover, consistent with differential selection pressures. Second, the turnover in sex-biased status was largely unrelated to cross-tissue expression breadth, suggesting pleiotropy does not restrict evolution of sex-biased expression. Third, the fold-sex-biased expression, for both testis-biased and ovary-biased genes, evolved directionally over time toward higher values, a crucial finding that could be interpreted as a selective advantage of greater sex-bias, and sexual antagonism. Fourth, in terms of protein divergence, genes with LSTs to testis-biased expression exhibited weak signals of elevated rates of evolution (than ovary-biased) in as little as 5 My, which strengthened over time. Moreover, genes with clade-wide testis-specific expression (44 My), a status not observed for any ovary-biased genes, exhibited striking acceleration of protein divergence, which was linked to low pleiotropy.

**Conclusions:**

By studying LSTs and clade-wide sex-biased gonadal expression in a multi-species clade of Drosophila, we describe evidence that interspecies turnover and magnitude of sex-biased expression have been influenced by selection. Further, whilst pleiotropy was not connected to turnover in sex-biased gonadal expression, it likely explains protein sequence divergence.

**Electronic supplementary material:**

The online version of this article (10.1186/s12862-019-1377-4) contains supplementary material, which is available to authorized users.

## Background

Sexually dimorphic phenotypes are thought to result from differential gene expression between the sexes, as most genes are common to both male and female genomes [1]. Sex-biased gene expression, or upregulated transcription in one sex, has been widely reported in animals, including species of mammals, birds, fish, worms, insects, as well as outside of animals, in fungi and higher plants, with estimates indicating that from 10% to more than 90% of the genome can exhibit sex-biased transcription depending on methods and taxon [1–24]. Sex-biased expression is believed to have arisen to resolve sexual conflict and thus might largely reflect selection acting on processes within and between sexes [1, 2, 18]. Sex-biases could also be shaped by pleiotropic constraints [25, 26]. At present however, the factors underlying the evolution of sex-biased expression in metazoans remain largely unresolved, particularly the roles of sex-related selection and pleiotropy [3, 18, 19, 25, 27, 28].

Drosophila has served as a primary model system for the study of sex-biased gene expression in animals [1, 2, 4, 6, 10, 11, 14, 15, 29–38]. Research in Drosophila has often been conducted using whole males and females, typically from one or two species, and sometimes pooled with sexual tissues; the studies have repeatedly shown evidence of sex-biased gene expression, rapid protein sequence divergence of male-biased genes, and/or interspecies turnover in sex-biased expression in this genus [1–3, 14–16, 31, 33, 35]. Studies focused on the turnover in sex-biased expression status in multiple Drosophila species has been relatively uncommon [8, 11]. This type of multi-species research, which has typically been conducted using gene expression data from whole males and whole females, has unveiled patterns such as enhanced interspecies variation in sex-biased expression as species diverge (e.g., increased standard deviation in ratios of female:male expression), a preference for male-biased genes to exhibit elevated gene losses or gains [8], correlations between expression and protein divergence [8], and concurring evidence for the widely observed pattern of rapid protein sequence divergence of male-biased genes in this taxon [1, 8, 11].

Crucially however, it has been widely thought, and in some cases empirically shown, that most of the expression differences between males and females in Drosophila, as well as other insects, originate from the sex-organs [3, 8, 14, 22, 24, 33]. In this regard, the study of gene expression from whole organisms could lead to an imprecise picture of sex biases [22, 35] due to dilution (allometric effects) of expression differences from the sex-limited tissues [22, 35, 39]. For such reasons, growing studies have focused on sex-biased expression in gonads in insects [14, 22, 24, 35], and in other models such as birds [19]. Further research in Drosophila using multi-species contrasts specifically of the gonadal tissues, and using original analytical approaches, thus provide a pathway to gaining further insights into how sex-biased expression evolves over time, and the factors shaping rates and patterns of turnover in Drosophila.

Studies on sex-biased gonadal expression in multi-species clades outside of Drosophila been reported only sporadically in the literature. As an example, a study of six species in the fowl clade Galloanserae reported that testis- and ovary-biased genes exhibited marked turnover (gains/losses) in sex-biased expression status between species in the phylogeny. This phenomenon was proposed to be linked to sexual selection, and was affirmed by testing the hypothesis in males, yielding findings of a positive association between male sexual ornamentation and turnover of testis-biased expression in the terminal species branches [19]. Further, species-specific transitions to testis-biased expression were primarily caused by increased expression in the male gonads, whereas those transitions to ovary-biased expression were often caused by down-regulation in testes [19]. This suggests that the testis largely control both testis and ovary sex biases in expression within those birds [19].

Another multi-species study was conducted in four species of cichlids [40]. That assessment showed that sex-biased gonadal expression profiles were conserved for a majority of genes studied in that genus [40]. However, there were some exceptions, such as for sex steroid genes, where sex-biased expression appeared to have shifted extensively among species [40]. In insects, an analysis of four species clade of Anopheles mosquitoes that included some gonadal comparisons, suggested rapid changes in sex-biased expression in that taxon. For example, interspecies expression divergence (standard deviation of male:female expression ratios, similar to Zhang et al. 2007 [8]) was elevated for ovary-specific, testis-specific, and strongly testis-biased genes, as compared to unbiased genes within that genus [23]. Further, sex-specific gonadal expression was connected to genic gains/losses [23]. Each of these multi-species studies has shown that sex-biased expression status has changed in a substantive manner in closely related taxa.

An important topic that should be considered when studying the evolution of sex-biased gene expression is the role of pleiotropy. For instance, studies in vertebrates have suggested that pleiotropy, measured as expression breadth across tissues, may act to restrict evolution of sex-biased gene expression [25, 41]. Furthermore, pleiotropic functions across multiple tissues may act to limit evolution of expression changes within specific tissues such as the gonads [8, 25, 26, 42]. That is, when genes are involved in multiple processes and/or tissues, they may have limited freedom to evolve changes in their sex-biased gene expression status within the reproductive organs [8, 25, 42]. Empirical assessment of the role of pleiotropy in the evolution of sex-biased gene expression *per se*, however, has only rarely been attempted to date *e.g.* [25, 26, 42]. Accordingly, pleiotropy should be considered as part of an assessment of the evolution of sex-biased gene expression.

A parameter that has been widely studied in conjunction with sex-biased gene expression in metazoans is protein sequence divergence, particularly the level of selective constraint acting on protein evolution (measured using the ratio of nonsynonymous to synonymous substitutions, dN/dS). It has been often reported, but with some exceptions (see for examples [22, 23, 43, 44]), that male-biased and/or male reproductive genes (including testis-biased, seminal fluid and/or sperm genes), evolve rapidly and/or exhibit positive selection as compared to female genes and/or the rest of the genome in Drosophila and other models [1, 4–6, 10, 14, 19, 30, 31, 45–48]. This is thought to potentially arise from sexual selection, including sperm competition, a notion consistent with some findings of positive selection in sex-related genes [1, 6, 10]. It has also been proposed, however, that the rapid evolution of male-biased genes might result from low pleiotropy and relaxed functional constraint that acts to accelerate protein sequence evolution [1, 8, 14, 16, 41]. In this regard, protein sequence evolution is an important factor to include in the study of the study of turnover in sex-biased transcription.

While Drosophila has served as a core invertebrate system for the study of sex-biased gene expression, additional study of this taxon using multi-species contrasts of the sex-organs, and employing atypical approaches, may help further decipher the dynamics shaping evolution of sex-biased transcription. Here, we rigorously assess the evolution of sex-biased gonadal gene expression in 10,740 genes across four species from the melanogaster group of Drosophila. For this, we identify genes with lineage-specific transitions (LSTs) in sex-biased status (SBS), standardized to the number of genes with the ancestral state (S-LSTs), and those with clade-wide sex-biases. We subsequently evaluate the patterns of turnover and magnitude of sex-biased expression over time, cross-tissue expression breadth, and the evolution of protein sequences. Together, the results provide insights into the roles of selection and pleiotropy in the evolution of sex-biased gonadal expression in this genus.

## Methods

Four species of Drosophila from the melanogaster group were used to assess sex-biased expression in the gonads: the well-established reference model *Drosophila melanogaster* and its sister species *D. simulans*, *D. yakuba* and *D. ananassae* (abbreviated as Dmel, Dsim, Dyak, and Dana hereafter). The phylogenetic relationship between these species is (((Dmel,Dsim),Dyak),Dana) and is shown in Additional file 1: Figure S1. The complete CDS sets per species were obtained from http://www.flybase.org [49] and versions are provided in Additional file 1: Table S1. The full CDS (longest isoform) per gene for each species was identified and used for study. Orthologs across the four species was determined using the ortholog database at www.flybase.org. [49], which provides high confidence orthologs to the reference Dmel for each of the three sister species studied here. Dmel genes that matched more than one ortholog in any compared species, or vice-versa, was excluded from analysis, such that all orthologs under study were one-to one matches. As the ingroup species Dmel contains the most well annotated and intensively studied genome in Drosophila, it is used as the reference system throughout our analyses.

### Expression profiling

For expression profiling, large-scale testis and ovary RNA-seq data were obtained from the SRA database for each of the four species under study (> 42 million paired-end reads per sample, Additional file 1: Table S1) [50]. The samples, as described at the SRA database, are testes and ovaries from virgin males and females dissected within 2–4 days after eclosion, and all specimens per species were grown, maintained and collected under the same conditions (see SRA project ID for details, and Ref. [50]; Table S1). The frequency per kilobase million (FPKM) was determined by mapping the reads per CDS in the Geneious Read Mapper in Geneious 11.0.3 (https://assets.geneious.com/documentation/geneious/GeneiousReadMapper.pdf). The program was run with two iterations [51]. To ensure precise read-CDS matches, the entire CDS list for each species was used for matching reads to the CDS, and then the results for the sub-set of genes with four-species orthologs were extracted and used for analysis. For genes without orthologs, we conducted a separate analysis of sex-biased expression in the reference Dmel.

We performed clustering analysis of gene expression levels across testes and ovaries for all four species. Hierarchical clustering of expression levels was conducted using Spearman’s correlations with average linkage in the program Cluster 3.0 [52], and output was visualized in TreeView (http://bonsai.hgc.jp/~mdehoon/software/cluster/software.htm).

### Identification of sex-biased genes

Sex-biased gene expression was defined for genes that exhibited at least a two-fold difference in expression cf. [6, 14, 22, 34] between testes and ovaries which was statistically significant (*P* < 0.05) and had a FPKM> 1 in at least one gonadal tissue. Thus, the definition of sex-biased expression herein is based entirely on differential expression in the gonads (see our “Assessments of Pleiotropy” section for nongonadal analysis). All genes not matching these criteria were defined as unbiased. Differential expression for each gene and *P*-values were determined using Geneious 11.0.3 [51], wherein the expression data per sample was normalized by the median and compared using its method designed for two sample contrasts of large-RNA datasets, and that employs the Binomial distribution in ascribing probability values per gene. As we had a high cutoff for the definition of sex-biased genes (two-fold minimum) and deep RNA-seq datasets (Additional file 1: Table S1) [50], this approach provides effective detection of differentially expressed genes amongst disparate tissues. The vast majority of genes (> 98.0% per species) with two-fold bias (or higher) and statistically significant differential expression (*P* < 0.05), retained this same status after Bonferroni correction. We thus used the uncorrected values for identification of sex-biased genes as we had a high threshold for defining a gene as sex-biased (≥2-fold difference), larger than applied in some other studies (*e.g.* 1.25-fold: [53]), and we wished to include all genes with a propensity for sex-biased expression. Only six of the genes studied exhibited no expression in any species; these were included in unbiased gene sets and their inclusion/removal resulted in the same findings in our study.

Testis- and ovary-specific genes were defined throughout as those sex-biased genes that had 0 FPKM in the opposite sexed gonad (i.e. from inter-gonadal contrasts). To affirm stringency in this assessment, the identified sex-specific gene sets were confirmed for the reference Dmel using the modENCODE RNA-seq database available for this species at FlyBase (http://www.flybase.org, http://www.modencode.org) [49, 54].

### Evaluation of lineage-specific transitions (LSTs) in sex-biased status

The few available studies of male- and female-biased expression turnover in two or more species of Drosophila have suggested an uncommonness of reversals in sex-bias, a trend not observed in birds [19], and/or some variation in gains/losses of sex-biases [3, 8, 11, 14]. To address this issue, we aimed to rigorously assess the rates of turnover in sex-biased status in each of the four species branches of Drosophila, with known divergence times [55], in a study design based on the following approaches: 1) examination of expression data strictly from the gonads; 2) evaluation of the number of genes with each of the six types of lineage-specific transition (LSTs) in sex-biased expression (among testis-biased, ovary-biased and unbiased status) and clade-wide conserved sex-biases and; 3) standardization of the frequency of each type of LST to the number of genes with the ancestral state (S-LSTs). This approach has several advantages. The S-LSTs allow comparison of turnover rates for each of six transitional categories, yielding an informative profile of putative differential selective pressures. In addition, the design permits an assessment of how the degree (fold-bias) of sex-biased expression has evolved over time (My), that is, between genes with LSTs and those with long term sex-biases (clade-wide). The approach also provides a means to assess any time-effects of conserved sex-biased, or sex-specific, gonadal status on dN/dS (i.e., in short versus long branches). Moreover, the LSTs (combined with cross-tissue expression breadth analysis) provide a novel means to assess whether pleiotropy acts to restrict transitions in sex-biased gonadal expression.

For the identification of LSTs, we identified genes that had a conserved ancestral sex-biased status (SBS) in three species and that had transitioned to a different SBS in the (fourth) lineage (terminal branch). As each terminal species branch represents a single period of divergence from its last common ancestor, we could thus compare the relative frequency of different types of transitions within each branch. We considered each of the six categories of LSTs in SBS (ancestral state to derived state), as follows: ovary- to testis-biased (ov-ts), unbiased to testis-biased (unb-ts), testis- to ovary-biased (ts-ov), unbiased to ovary-biased (unb-ov), testis-biased to unbiased (ts-unb), and ovary-biased to unbiased (ov-unb) expression. As the frequency of LSTs observed per branch will depend on the number of genes (N) that had the conserved ancestral state when the branch diverged, we standardized LSTs (S-LSTs) in each target branch as follows: S-LSTs_(x-y target branch)_ = N_LSTs(x-y target branch)_ / (N_SBSx (the same clade-wide SBS in all 4 branches)_ + N_LSTs(x-y target branch) +_ N_LSTs(x-z target branch)_) X 1,000, where x = the ancestral state (e.g., unb), y = derived state in the target branch (e.g., x-y = unb-ts), z = the alternate type of transition from the ancestral state in the target branch (e.g., x-z = unb-ov) and N_SBSx_ = the number of genes with the same ancestral (x) sex-biased status in all four species. We multiplied by 1,000 for ease of readability and interpretation. The denominator controls for the number of genes with the conserved ancestral state that were available for a specific type of SBS transition within the target branch, and thus S-LSTs are comparable across transition types. We specify that the LSTs in Dmel, Dsim, and Dyak all indicate a gain in branch-specific status (e.g., ov-ts indicates a gain in testis-bias in one branch), while for the outgroup, Dana, a lineage-specific SBS would typically indicate a gain in lineage-specific status, but might sometimes comprise a reverse change in SBS within the relatively short branch from the common clade node to the ingroup clade (Dmel, Dsim and Dyak). We included the S-LSTs for Dana given the status is lineage-specific under either scenario, and conservatively note this caveat for interpretation of Dana S-LSTs (note: see later section “*Directional increase in fold sex-biased expression over time*”). The determination of S-LSTs was repeated for all four species branches and all six categories of transitions.

### Assessments of pleiotropy

Pleiotropy denotes a gene’s multi-functionality across various genetic pathways or processes. Expression breadth comprises a proxy of the range of functions of a gene across various tissues or stages, and thus provides a measure of a gene’s pleiotropy [25, 31, 41]. To assess expression breadth, and thus pleiotropy, of genes under study here, we used large-scale transcriptome data available for the well-studied and reference model species Dmel in modENCODE as available at FlyBase [49]. From these data, we determined expression breadth as the percentage of genes exhibiting the presence of expression across the 17 disparate developmental stages and tissues described in Additional file 1: Table S2. In turn, we determined the association between expression breadth and S-LSTs for Dmel (such that expression breadth data and S-LSTs were from the same species) and to dN/dS for genes with clade-wide sex-biased status.

### Protein sequence divergence

In order to assess protein sequence divergence, CDS for all four orthologs per gene were aligned at the codon level using Mega-CC [56] and default settings with the exception that the gap penalty was set at −1.9. Subsequently, the dN /dS values per terminal branch were determined using maximum likelihood in the codeml package in PAML under the free-ratio (M1) model and the unrooted tree [57]. This model allows dN/dS to vary among branches and determines an independent dN/dS value for each terminal species branch, that are needed for our branch-specific assessments. Values of dN/dS < 1, =1 and > 1 indicate a prevalence of purifying selection, neutral evolution and positive selection respectively [57]. However even when < 1, elevated dN/dS values indicate accelerated evolution, which could be due to relaxed constraints and/or adaptive changes. As a conservative approach, for analysis of each species’ terminal branch, we examined only genes in that branch with values of dS and dN below 1.5 (and dS > 0.001) including for the relatively distant outgroup Dana (for further details, see Additional file 1: Text File S1). This range of dN and dS values represents levels effective for limiting saturation of substitutions and ensuring quality of sequence alignments [58].

Positive selection was tested using “sites” analysis in PAML [57] using all four species Dmel, Dsim, Dyak and Dana by comparing models M7 versus M8 [57]. We conducted this assessment for all genes with clade-wide sex biased status. Branch-site analysis was conducted for those genes exhibiting LSTs from unbiased to sex-biased status (unb-ts and unb-ov) using the branch with an LST as the tested branch [57]. *P*-values < 0.05 for each analysis were determined using 2∆lnL and the Chi^2^-distribution as described in the PAML manual [57]. Results for positive selection analyses include only genes where all taxa (sites analysis, or the branch of interest (branch-site analysis), had dN and dS < 1.5 and dS > 0.001), and thus are conservative estimates.

For additional rigor, we obtained the dN/dS values for the genes under study from the flyDIVaS database, which has values for a six-species group in Drosophila, comprising the four species studied herein plus *D. sechellia* and *D. erecta* [59]. The database has determined dN/dS values using the M0 model in PAML, which unlike M1 (which we used herein to obtain species-specific values), provides a single dN/dS across all species branches. We compared the dN/dS values from our assessment (using mean dN//mean dS across four species branches) to the M0 values from flyDIVaS and tested a correlation using Spearman’s ranked R. In addition, we compared our positive selection tests for M7 versus M8 to those available at flyDIVaS.

### Functional analyses

For gene functional analysis, all gene ontology (GO) assessments were conducted using the GO clustering program DAVID [60]. Gene functions were assessed using the identifiers from the well annotated and reference species Dmel which are accepted in DAVID for functional analyses of genes.

### Availability of data and materials

The protein-coding DNA sequences for all genes studied within each of the four Drosophila species, Dmel, Dsim, Dyak and Dana, are available publicly at FlyBase [49]. Version numbers are provided in Additional file 1: Table S1. The between species ortholog datasets are also available at FlyBase [49]. The RNA-seq data are available at the SRA database under identification numbers in Additional file 1: Table S1 [50]. The flyDIVaS data are available as described in [59]. All data used for this study are public.

## Results

Of the 13,933 annotated protein-coding genes from the reference Dmel genome, we identified 10,740 (77.1%) that had one-to-one orthologs in Dsim, Dyak and Dana. For consistency, all our main analyses of sex-biased expression were conducted using these 10,740 orthologous genes. Findings for the genes that lacked orthologs are described in the later section “*The genes without orthologs exhibit testis-biased expression*”. Divergence times from Dmel to the last common ancestor with each of Dsim, Dyak and Dana have been reported as 5, 13 and 44 Mya respectively [55].

### Descriptive summary of the sex-biased gene sets

**Fig. 1:**
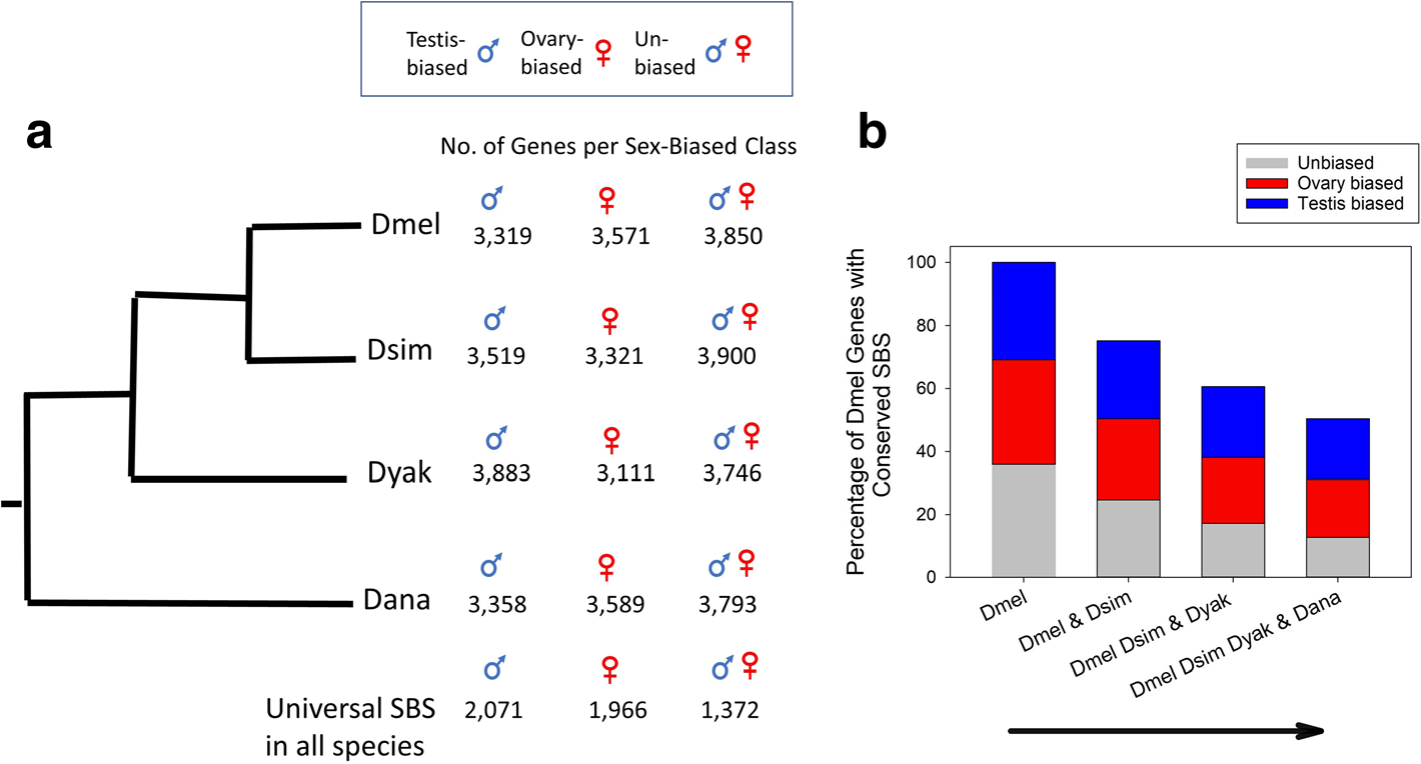
Sex-biased gonadal expression in four Drosophila species**. a**) Schematic diagram of the number of testis-biased, ovary-biased and unbiased genes for each species under study. The number of genes with universal (clade-wide) sex-biased status (SBS) in all four taxa are also shown. **b**) Using the ingroup taxon *D. melanogaster* as the reference, the percentage of testis-biased, ovary-biased and unbiased genes (among 10,740 genes) that retained conserved SBS with each step towards the outgroup (*D. ananassae*) in the phylogeny. The number of genes with conserved testis-biased, ovary-biased and with unbiased status was statistically significantly lower between each step in the phylogeny (for each category between steps Chi^2^ *P* < 0.0001)

Before our main analyses, we first describe here the properties of the sex-biased gene sets under study. Of the 10,740 genes with orthologs, nearly all were expressed in one or both gonads in each of the four Drosophila species (between 97.7 and 98.7% depending on the species). Using our criteria of ≥2-fold bias (*P* < 0.05) to identify sex-biased genes, we found that more than 63% of the 10,740 genes studied were sex-biased in each species (Fig. 1a). Specifically, 6,890 (64.2%), 6,840 (63.7%), 6,994 (65.1%) and 6,947 (64.7%) were sex-biased for Dmel, Dsim, Dyak and Dana respectively.

Extensive turnover was observed in testis-biased and ovary-biased status across the four species. In particular, 5,331 (49.6%) of the 10,740 studied genes exhibited variation in SBS across species, while 50.4% (5,409) had the same clade-wide (universal) SBS in all four species (Fig. 1a). In other words, for half of the genes studied, the sex-biased expression status differed in at least one species as compared to the others. As shown in Fig. 1b, using the ingroup species Dmel as the reference, the proportion of genes which had conserved testis-biased, ovary-biased or unbiased expression declined in a stepwise manner with increasing divergence towards the outgroup, that is, from Dmel to Dsim (conserved in both), to Dyak (conserved in three species) to Dana (conserved in all four species; Chi^2^ *P* < 0.0001 for contrasts of the number of testis-, ovary-, and of unbiased genes between each step, Fig. 1b), a pattern concurring with monotonic changes in SBS observed in whole files [8]. At the clade-wide level, a total of 2,071 (19.2% of 10,740), 1,966 (18.3%) and 1,372 (12.8%) genes respectively retained the same testis-biased, ovary-biased and unbiased expression universally across all four species (Fig. 1a).

#### Testis- and ovary-specificity of expression

Testis-specific expression, defined herein as those testis-biased genes with zero expression in ovaries, were much more common in the testis-biased gene sets (varying between 13.86 and 34.90% of the testis-biased gene sets depending on the species, see Table 1), than were ovary-specific genes in the ovary-biased gene sets (between 0.18 and 0.48%, Chi^2^ test between testis- and ovary-biased genes per species *P* < 0.0001, Table 1). This suggests greater specialization of functions in the testis-biased genes. Furthermore, 171 genes exhibited clade-wide testis-specificity (relative to ovaries) in all four Drosophila species, whilst no ovary-specific genes had clade-wide status. The finding that testis-specificity is more common than ovary-specificity expression in all four of these Drosophila species is in agreement with expression studies in the single species Dmel [33, 39] and in other species such as wasps [21]. Our present results show that testis-specificity has been strongly conserved at the clade-wide level for a major subset of testis-biased genes over a period of 44 My, a status not observed for any ovary-biased genes.

**Table 1:** The frequency of genes with sex-specific expression in testes and ovaries in each species of Drosophila**.** The percentage of the sex-biased gene sets represented by sex-specific genes (zero expression in the opposite sexual organ) is shown. The number of clade-wide testis-specific genes, which were specific in all four species, was 171 and there were no clade-wide ovary-specific genes. Chi^2^ tests of the percentage of testis- versus ovary-specific genes per species *P* < 0.0001 for each species. Sex-biased expression and sex-specificity was determined by inter-gonadal contrasts

Category	No. of Genes per Species								
	Dmel		Dsim		Dyak		Dana		Clade-Wide Specificity
	N	Percent^a^	N	Percent	N	Percent	N	Percent	N
No. of Testis-Specific Genes	692	20.85	870	24.72	538	13.86	1,172	34.90	171
No. of Ovary-Specific Genes	17	0.48	6	0.18	9	0.29	8	0.22	0

^a^The percent of testis-biased or ovary-biased genes that were testis- or ovary-specific respectively

For additional rigor, we further assessed the specificity of expression of the 171 genes with clade-wide testis-specificity (which are listed in Additional file 1: Table S3) using expression profiles available for Dmel at the modENCODE Anatomy RNA-seq database (mated testes and ovaries, flybase.org; http://www.modencode.org), and found strong concordance with our results. Specifically, 100% (*N* = 171 of 171) of the testis-specific genes identified herein were observed in modENCODE as exhibiting expression in the testes, and as having no detectable transcript reads in ovaries, thus affirming the high accuracy of the RNA-seq dataset(s) (Table S1) and methods used herein for discerning sex-specific expression profiles.

#### Expression level divergence is higher in testes than ovaries

**Fig. 2:**
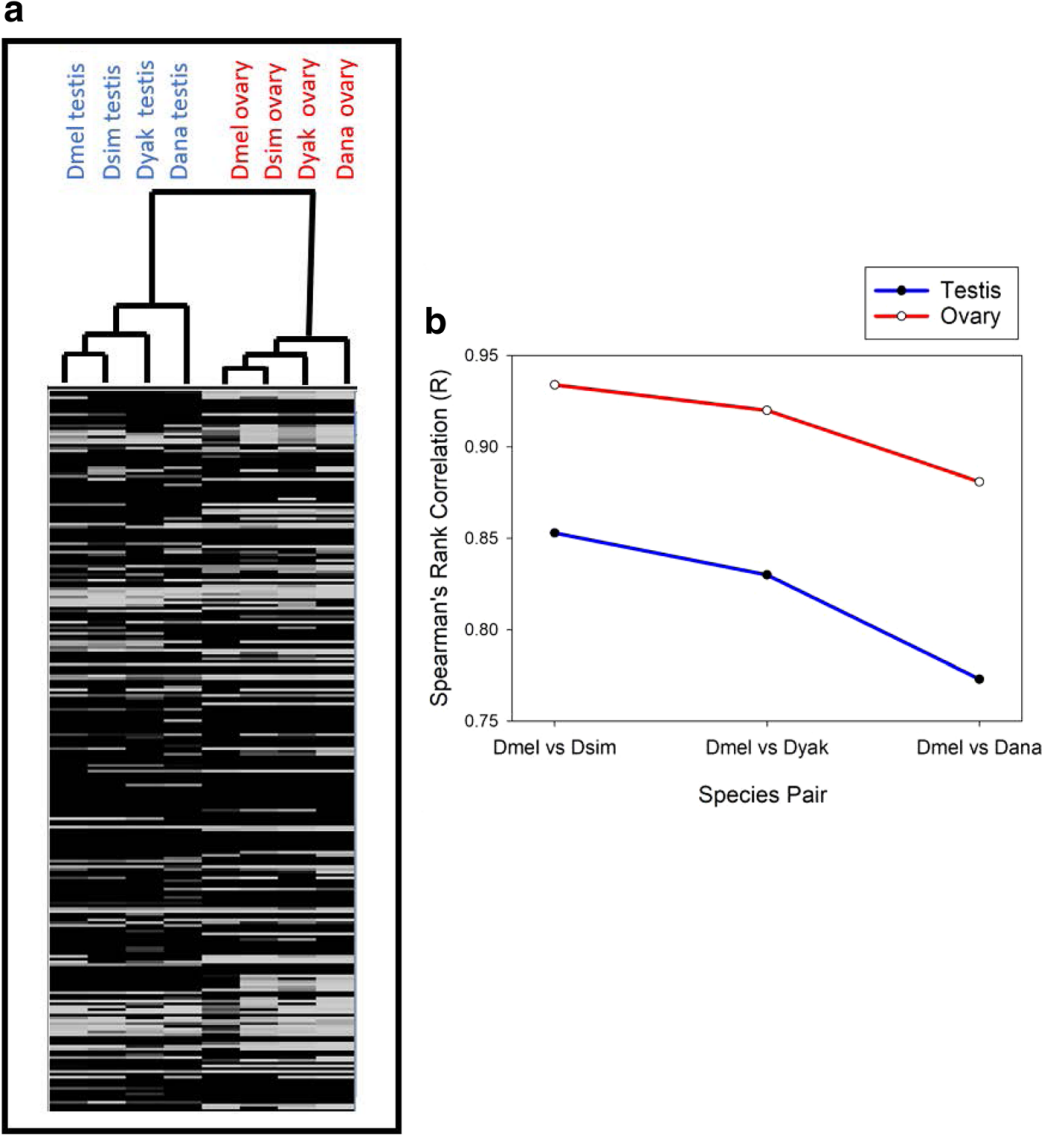
Interspecies divergence of gene expression level of testis and ovary profiles in four species of Drosophila**. a**) Hierarchical clustering of expression level. The heatmap shown comprises a representative sample of all genes under study (genes with FPKM≥1 included); **b**) The Spearman R correlations for expression level (FPKM) of Dmel versus (vs) the other three species in the phylogeny (*P* < 2.0X10^− 7^ for each contrast; all genes included). Species names are abbreviated as described in the main text

We assessed the interspecies divergence in gene expression level per se, that is the changes in expression (FPKM) for genes transcribed in the testes and for the ovaries. Hierarchical clustering of gonadal expression levels across all genes (with FPKM> 1 in at least one gonad), showed that expression level clustered primarily by sex, and secondarily by phylogenetic relatedness (Fig. 2a). As indicated in Fig. 2a, the gene expression of testes and ovaries formed separate groups that each contained Dmel, Dsim, Dyak and Dana, showing that gonad expression, rather than phylogenetic relatedness, is the primary factor shaping expression profiles. Whilst few studies to date have conducted comparable analyses of expression in the gonads in a multi-species clade, we note that this result concurs with results for a phylogeny of six bird species (Galloanserae) [19], suggesting that a strong gonadal-expression effect may be common to divergent animal systems. The clustering of gonadal expression by sex, rather than by phylogenetic relatedness (Fig. 2a), may be deemed consistent with a history of sex-related selection, such as purifying selection and/or sexual selection [19].

Gene expression levels (FPKM) within the testes and within the ovaries has diverged substantially among the four Drosophila species. Specifically, using the ingroup Dmel as the reference species (Additional file 1: Figure S1), we determined the interspecies rank correlations in expression level. As shown in Fig. 2b, testis-expression level (across all genes under study) was strongly correlated between taxa, with Spearman’s R for Dmel versus Dsim R = 0.85, Dmel versus Dyak R = 0.83 and Dmel versus Dana R = 0.77 (*P* < 2X10^− 7^ per species pair). Similar trends were observed for ovary expression (R = 0.93, 0.92, and 0.88 respectively, *P* < 2X10^− 7^). The progressive decline in correlation coefficients from Dmel versus Dsim, to Dmel versus Dyak to Dmel versus Dana, is consistent with greater divergence of testis- and ovary-expression levels (FPKM) with greater time, similar to divergence patterns observed for whole males and females [8]. Further, the between-species R values observed here for ovary expression were consistently higher than the comparable values for testis expression, indicating accelerated interspecies divergence in testis- versus ovary-expression.

### Evolution of standardized lineage-specific transitions in SBS

For our main assessments herein, we examined genes with LSTs in SBS across the phylogeny and those with clade-wide biases. LSTs are transitions from a conserved ancestral sex-biased state (shared amongst three species) to a derived status in a single (fourth) terminal branch and were standardized to the number of genes with the ancestral state (S-LSTs) for each of the six transition categories. Using these data, we conducted several analyses including assessment of differential S-LSTs levels among transition types per species, the relationship between S-LSTs and pleiotropy, and how the magnitude of (fold-) sex-biased expression evolved in genes with LSTs evolved over time.

As shown in Table 2, we found that of the 5,331 genes exhibiting variation in SBS (Fig. 1a), 64.1% (3,431 genes) had an LST in SBS in the Drosophila clade (Additional file 1: Table S4). The S-LST values per transition category per species are shown in Table 2. We report that the most uncommon transition in all four species was ov-ts, which had S-LST values of 1.9 up to 13.9 transitions (per 1,000 genes with ancestral ovary-bias) in Dmel and Dana respectively. For comparison, unb-ts transitions were between 8.0 to 48.1-fold more frequent depending on the species branch (Chi^2^ *P* < 0.0001, Table 2), inferring most transitions to testis-biased expression arose from an ancestrally unbiased state. The next least common type of transition in each species branch, with S-LST values between 5.0 and 21.5 was ts-ov. The unb-ov transitions were between 4.4- and 15.5-fold more common than ts-ov depending on species (*P* < 0.0001 for all four species branches). Thus, despite species-specific variation in the scale of the effect, all four species branches indicate that sex-biased expression more commonly originated from an unbiased state (unb-ts, unb-ov) than from reversals (ov-ts, ts-ov) in SBS.

**Table 2:** The standardized-lineage-specific transitions (S-LSTs) in sex-biased status (transitions between testis-biased, ovary-biased or unbiased status) in the gonads from a conserved ancestral state. The six possible categories of transitions in sex-biased expression are shown per species. Comparison of the ratio of S-LSTs are shown. *Indicates a Chi^2^ test between the two transition types per contrast (using counts of transitions and ancestral states) was statistically significant (*P < 0.05 and ≥ 0.0001, **P < 0.0001). Raw counts of transitions are in Additional file 1: Table S4

Type of Transition (Ancestral to derived)	Standardized (S)- LSTs			
	Dmel	Dsim	Dyak	Dana
*Gain of Testis-Bias*				
Ov-Ts	1.92	2.32	3.43	13.85
Unb-Ts	90.26	111.64	163.72	111.11
*Ratio Unb-Ts* vs *Ov-Ts*	47.14**	48.14**	47.68**	8.03**
*Gain of Ovary-Bias*				
Ts-Ov	21.47	15.17	4.99	19.06
Unb-Ov	95.01	69.25	77.43	148.87
*Ratio Unb-Ov* vs *Ts-Ov*	4.42**	4.56**	15.51**	7.81**
*Loss of Sex-Bias*				
Ts-Unb	106.53	60.69	54.93	122.67
Ov-Unb	56.97	85.81	152.79	108.08
*Ratio Ov-Unb* vs *Ts-Unb*	0.53**	1.41*	2.78**	0.88
*Ratio of Reversals in Sex-Bias*				
Ts-Ov vs Ov-Ts	11.18**	6.54**	1.45	1.38**

Significantly, while the results in Table 2 indicate that S-LSTs for reversals were relatively uncommon events in each of the four Drosophila species, as suggested from some studies of males-females [3, 8, 11], they also importantly reveal that there are marked differences in S-LSTs of the two types of reversals in sex-biased status. For instance, ts-ov transitions were consistently more common than ov-ts transitions in all species (1.4 to 11.2 fold more common), particularly in the more recently derived branches for Dmel and Dsim (was statistically significant for in all species Chi^2^ test *P* < 0.0001, except Dyak *P* > 0.05). Thus, our results here using S-LSTs in gonads expose a significant difference between reversal types and suggest ts-ov transitions may be apt to be more beneficial (or less deleterious) than the ov-ts transitions at the interspecies level (for functions of genes with these LSTs see Additional file 1: Table S5).

With respect to losses in sex-biased status, that is, S-LSTs from testis- or ovary-biased status to an unbiased status, values varied to some extent among species. Specifically, we found S-LSTs arose at a similar level from an ancestrally testis-biased (ts-unb) and ovary-biased (ov-unb) state for Dana (*P* > 0.05). For Dsim and Dyak, ov-unb transitions were 1.4- and 2.8-fold more common than ts-unb respectively (Chi^2^ *P* < 0.0001 for each contrast), with the opposite trend found for Dmel where ts-unb was 1.9-fold more common (Chi^2^ *P* < 0.0001). Although ov-unb LSTs could be expected to be more common than ts-unb simply because ovary-biased genes typically had lower fold sex-bias (than testis-biased; see section “*Directional increase in fold sex-biased expression over time*”), and thus more genes were near the threshold of ovary-biased and unbiased status (two-fold cutoff applied herein) [8], we found no consistent pattern of an effect across species in Table 2. Most importantly, in all four Drosophila species the frequency of transitions from ts-unb was greater than ts-ov and ov-unb was greater than ov-ts (4.0- to 44.5-fold higher depending on the species; Chi^2^ *P* < 0.0001 for all branches). Thus, ancestrally testis-biased and ovary-biased genes were each much more likely to transition to an unbiased status than to convert to the opposite type of sex-biased expression.

An additional finding worth noting in Table 2 is that the rate of turnover in SBS varied among species branches. Based on divergence times of 5 My for Dmel and Dsim and 13 and 44 My for Dyak and Dana respectively [55], one might have expected a steady increase in S-LSTs over time, particularly between the two ingroup species Dmel and Dsim versus Dyak and Dana. However, that trend was only clearly observed for ov-ts and ov-unb, and not for the remaining categories. In this regard, these patterns also suggest that while neutral evolution likely contributes towards evolution of gonadal expression, non-neutral and/or species-specific pressures influence the rate of turnover in sex-biased expression.

The functions for genes with reversals in sex-biased expression and for those with unbiased to sex-biased transitions are shown in Additional file 1: Tables S5-S7. One noteworthy pattern is that genes with LSTs from unbiased to an acquired testis-biased status were convergently linked to olfactory functions in all four Drosophila species (Additional file 1: Tables S6-S7). This pattern appears similar to our prior findings for Aedes, wherein ovary-specific genes [22], which evolved faster than testis-biased genes in that taxon, were preferentially involved in olfactory functions [22]. Olfactory genes, in addition to their roles in attraction, have been linked to testis and sperm functions (including motility, sperm-egg attraction) in metazoans [61]. Thus, this result for LSTs to testis-biased status may infer evolution of new or expanded roles of olfactory genes in the male gonads after this transition.

Together, the patterns shown in Table 2 reveal marked differences in S-LSTs among the six transition types. The low S-LST values for reversals, and differences between the two types of reversals in gonadal sex-biases, are each consistent with a role of sex-dependent selection rather than (entirely) neutral evolution of expression [3].

#### Pleiotropy is unlinked to lineage-specific transitions in sex-biased expression

We next addressed whether pleiotropy could drive the patterns of turnover in sex-biased gonadal expression in Table 2. Genes expressed across multiple tissues have been shown to exhibit low interspecies expression divergence [42], a factor that may restrict evolution of sex-biased gene expression [25]. This topic has rarely been empirically addressed in the literature, and our method of studying S-LSTs provides an original means to tackle this issue. We assessed whether genes with LSTs for each of the six types of SBS transitions exhibited differences in expression breadth which might explain their frequency using the reference model Dmel (N total LSTs = 739, Additional file 1: Table S4). By analysing Dmel, wherein expression data are available for a wide range of tissues, and analyzing S-LSTs in this same branch (thus, expression data and LSTs are from the same species), we have the means to test any cause-effect relationship. For this, we used expression breadth across 17 disparate developmental stages and tissues (Additional file 1: Table S2) as a proxy for a gene’s pleiotropy [16, 25, 31, 41].

**Fig. 3:**
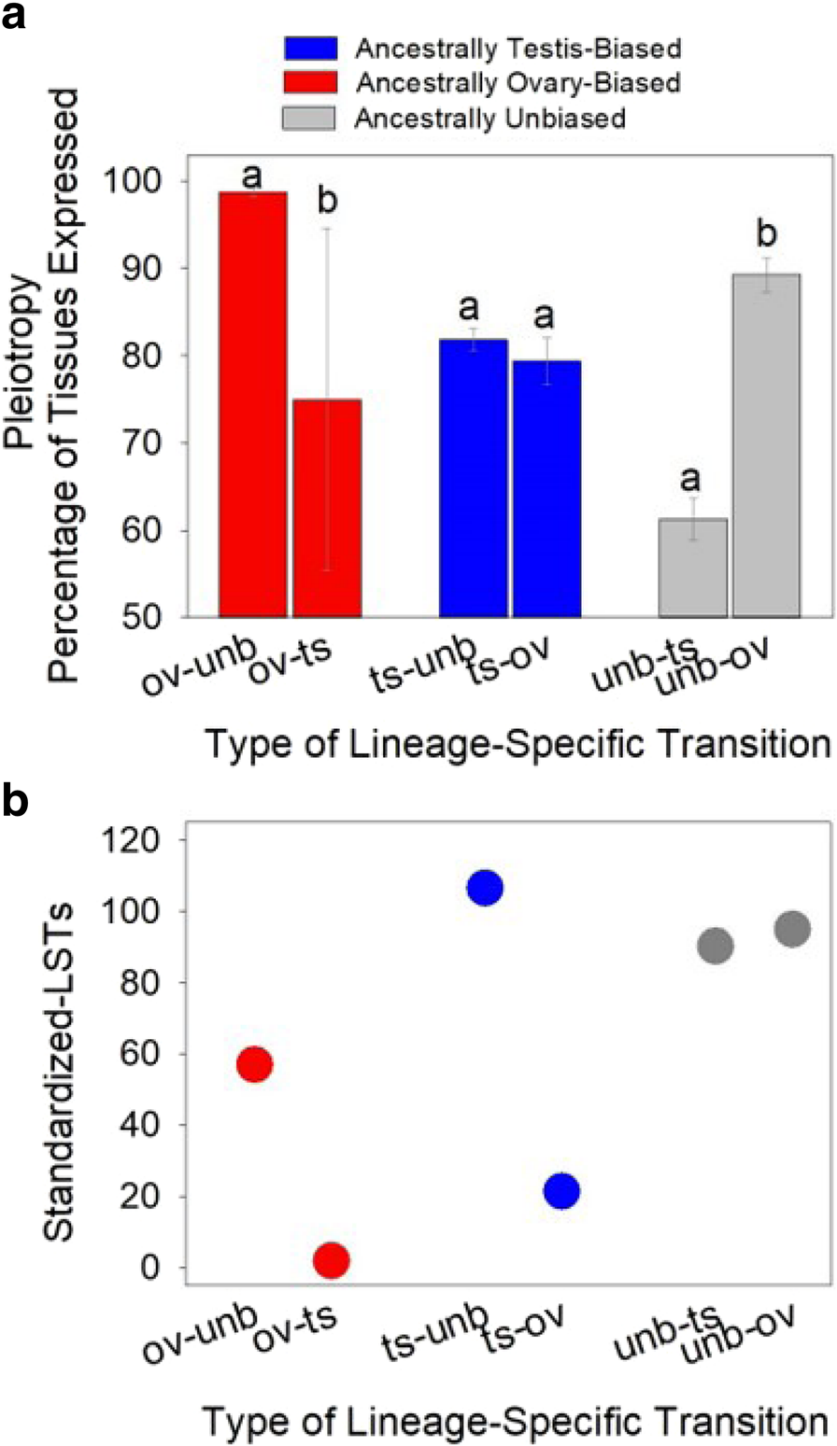
Expression breadth and the frequency of lineage-specific transitions (LSTs) in sex-biased status in *Drosophila melanogaster*. **a**) Average expression breadth for genes with LSTs for each of six types of transitions in Dmel and; **b**) the number of standardized-LSTs for each of six transition types for Dmel from Table 2. For **a** and **b** the transition types are listed on the X-axis in pairs with respect to the ancestral sex-biased state. Different letters above bars indicate a statistically significant difference within each pair using MWU-tests

The results, shown in Fig. 3, reveal that mean expression breadth was > 89% for genes with ov-unb and unb-ov transitions (98.72 ± 0.49, 89.22 ± 1.98), with lower values for ts-unb, ts-ov and ov-ts (81.82 ± 1.26, 79.25 ± 2.66 and 75.00 ± 19.57) and the lowest value for unb-ts (61.26 ± 2.44) (Fig. 3a). Pairing the transition types into those sharing the same ancestral SBS showed that expression breadth was not connected to the frequency of S-LSTs. For instance, much higher expression breadth was observed for genes with ov-unb than ov-ts (net difference in breadth > 23%, Fig. 3a, MWU-test *P* = 0.015), and yet the former transition type had a nearly 30-fold higher level of S-LSTs (Fig. 3b, Table 2, Chi^2^ *P* < 0.0001). In turn, for genes with ts-unb and ts-ov transitions, average expression breadth was in a similar range (79 to 82%, MWU-test *P* > 0.05, Fig. 3a), and despite this, the S-LSTs were five-fold higher for the former transition type (Fig. 3b, Chi^2^ P < 0.0001). These findings indicate that the elevated number of successful transitions from sex-biased to unbiased status as compared to reversals in sex-bias is not due to lower expression breadth (in the former group). Importantly, for the unb-ts and unb-ov categories, which comprised the majority of transitions to an acquired sex-biased status (Fig. 3b, Table 2), the former transition type had much lower expression breadth (net reduction of 28%, MWU-test *P* < 0.05), whilst both categories exhibited similar frequency of S-LSTs (90.3 and 95.0 respectively, Chi^2^ *P* = 0.90, Fig. 3ab, Table 2). This result suggests that the frequency of transitions from unbiased to an acquired sex-biased status were unrelated to pleiotropy. The differences in S-LSTs for the two types of reversals also cannot be explained by pleiotropy, as expression breadth was marginally higher for genes with ts-ov than ov-ts and yet the former had 11-fold elevated S-LSTs (Fig. 3b, Table 2). The losses in sex-biased expression to an unbiased state (not reversals), ts-unb and ov-unb, could perhaps suggest a relationship for those particular categories, where the former had lower expression breadth (MWU-test *P* < 0.05) and higher (1.9 fold) S-LSTs (MWU test-P < 0.05, Table 2, Fig. 3). Nonetheless, when taken collectively, while expression breadth varied among the genes with each of the six types of LSTs, it does not appear to be consistently linked to turnover in the sex-biased status within Dmel.

While pleiotropy has been thought to hamper the evolution of sex-biased gene expression [25, 41], the data in Fig. 3 for S-LSTs and expression breadth in Dmel indicates pleiotropy has not consistently restricted transitions in gonadal sex-biases in this taxon. Under a conservative interpretation, we note that the expression breadth in Dmel represents patterns observed in the extant species, and thus may have historically (in the Dmel branch) exhibited some variation. Further, categorical changes in SBS can be sensitive to methods and threshold cutoffs [8]. We therefore do not exclude any role of pleiotropy in shaping gonadal LSTs in this species.

#### Directional increase in fold sex-biased expression over time

**Fig. 4:**
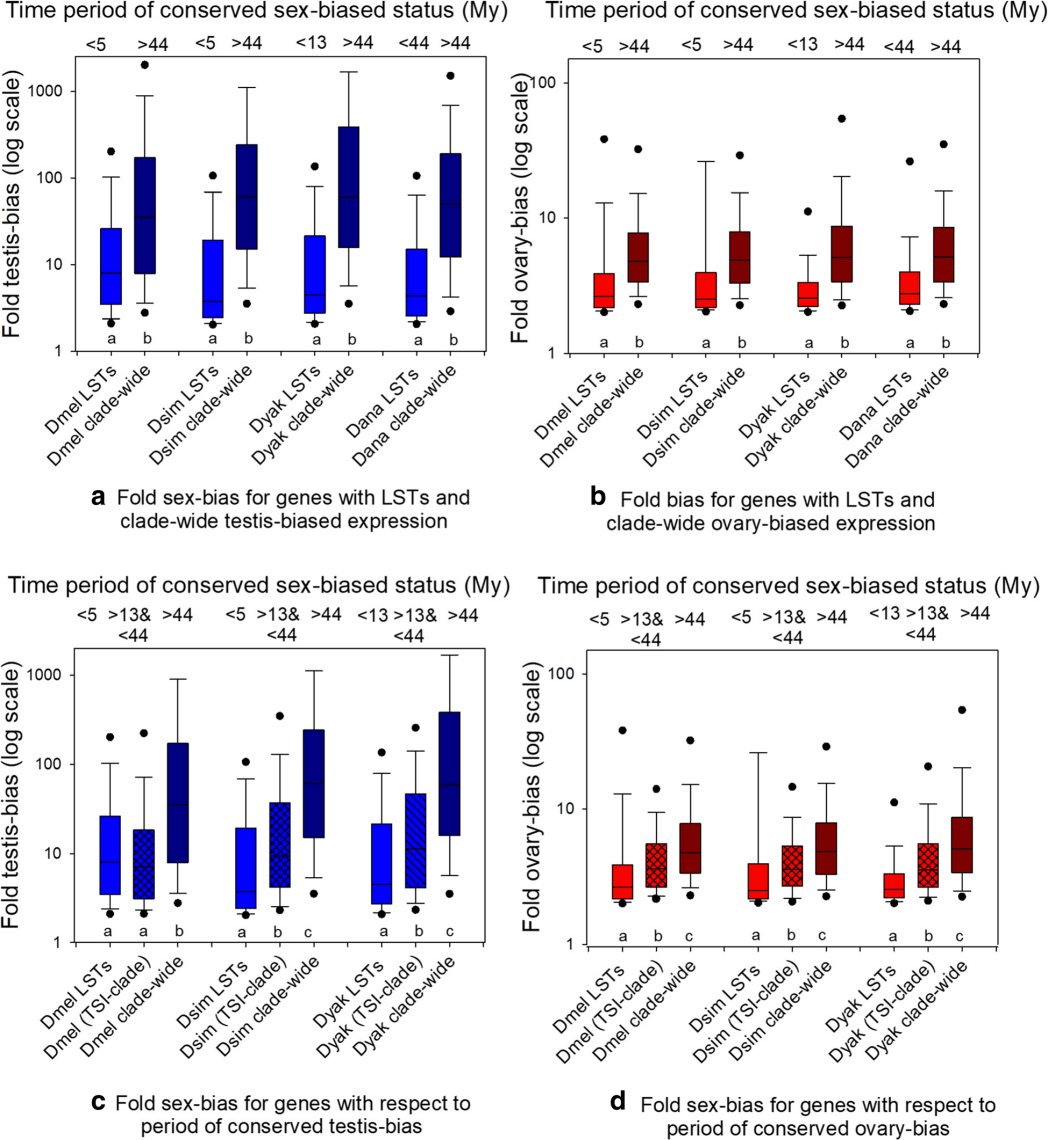
Box plots showing the distribution of the fold sex-bias for genes over time. **a**) Fold testis-biased expression for genes with lineage-specific transitions (LSTs) and with clade-wide testis-bias in four Drosophila species; **b**) the equivalent results to **a** for ovary-biased genes; **c**) fold testis-biased expression for genes with LSTs, with conserved bias in the three species ingroup (TSI) clade, and with clade-wide testis-biased expression; **d**) the equivalent results to **c** for ovary-biased genes. The minimum or maximum divergence time (My) is shown for each bar. Different letters below each bar per pair in **a** and **b** are statistically significantly different (MWU-*P* < 0.05) and in each triplet in **c** and **d** (Ranked ANOVA and paired Dunn’s test P < 0.05). The fold sex-bias for each of the eight testis-biased genes sets (eight bars) in panel **a** was higher than its ovary counterpart in panel **b** (MWU-P < 0.05). Note the Y-axes are in log scale and have different upper limits in panels. Clade-wide biases are shown in dark blue and red and LSTs with standard blue and red. TSI genes in panels **c** and **d** have crossbars

As part of our analyses of LSTs, we considered whether and how the magnitude of sex-biased expression evolved over time, that is, was fold sex-bias lower or higher for genes with LSTs versus genes with clade-wide (conserved) sex-biased status. For this, we assessed fold bias of genes with LSTs from unbiased to testis-biased expression and from unbiased to ovary-biased expression (unb-ts, unb-ov) in each species and genes with wide clade-wide sex-biases (Fig. 4a-d). We found that genes with LSTs from unbiased to testis-biased expression had markedly lower fold bias than genes with clade-wide testis-biased expression for each of the four species (Fig. 4a, MWU-test *P* < 0.05 for each type of transition in all species contrasts). In turn, as shown in Fig. 4b, genes with LSTs from unbiased to ovary-biased expression had statistically significantly lower fold-ovary biased expression than those with clade-wide ovary-biased expression (MWU-tests *P* < 0.05). Collectively, these findings demonstrate that without exception fold sex-bias was exceedingly weaker for genes with LSTs, that is those with branch-specific biases, than genes with clade-wide sex biases in expression, thus supporting a paradigm wherein the fold bias directionally increases over evolutionary time in a non-random manner.

We then examined those genes with conserved sex-biased status solely in the three-species ingroup (TSI) clade, of Dmel, Dsim and Dyak (that is, shared SBS in three species, and had a different status in Dana), which diverged 13 My, less than one third the time of those with clade-wide status (44 My) [55]. This analysis affirmed a pattern of a progressive increase in fold sex-biased status over time, from 5 My, to 13 My, and then to 44 My for testis-biased expression, and the same pattern was found for ovary-biased expression (Ranked ANOVA and Dunn’s contrast *P* < 0.05 for all contrasts per species for testis-biased genes (Fig. 4c) and ovary-biased genes (Fig. 4d); note that there was one exception, Dmel in Fig. 4c *P* > 0.05 in TSI versus LST genes).

The results in Fig. 4a-d explicitly demonstrate the existence of a phenomenon whereby fold sex-biased expression has increased over time and is observed in both testis-biased and ovary-biased genes. That is, an unambiguous and directional amplification in fold ovary- and in fold testis-bias with a greater time period of conserved sex-biased state. The striking progressive increase in the magnitude of testis-bias and fold ovary-biased expression over evolutionary time in Fig. 4a-d points towards a selective role in elevated fold sex bias. For instance, a neutral process acting on fold sex-bias may be anticipated to be non-directional (that is, not necessarily increasing fold sex-bias, but also decreasing fold sex-bias over time, and thus nondirectional), rather than strongly directional as found here (Fig. 4). Thus, the pattern infers sex-related processes, potentially inter-locus sexual antagonism [19, 27], could be involved in this phenomenon.

Several complementary results with respect to fold sex-bias are worth describing. The degree of fold bias of testis-biased genes in panel 4a was higher than observed for its parallel set for ovary-biased genes in panel 4b (MWU-test P < 0.05), indicating differences in fold bias between the sexes. Our results further show that the enhanced level of testis fold-bias (than ovary fold-bias) must have first arisen shortly after acquisition of testis- and ovary-biased expression, as the effect was even observed for LSTs to testis- and ovary-biased expression in Dmel and Dsim, which is less than 5 My (Fig. 4ab, MWU tests P < 0.05). Furthermore, we studied whether fold sex-bias was mostly controlled by testis- or ovary expression levels in each species. For this, fold bias was classified into three categories as has proven valuable for studying fold bias, ≥2- to 5-fold, ≥5- to 10-fold and ≥ 10-fold (N values per category provided in Additional file 1: Table S8) [20, 22, 35]. We show in Additional file 1: Figure S2 that fold testis-biased and fold ovary-biased expression depended on both expression levels in the ovaries and in testes. Thus, the degree of sex-biased expression was not controlled by changes in expression in one sex (see Additional file 1: Text File S2). Finally, we had noted (in our Methods) that for the outgroup species Dana some LSTs could have resulted from a reverse transition type in the shared ingroup branch (to Dmel, Dsim and Dyak). However, the fact that our Dana results on fold-bias (Fig. 4a,b) concurred nearly perfectly with each of the ingroup species suggests that many or most LSTs to testis-biased (Fig. 4a) or ovary-biased expression (Fig. 4b) occurred within the Dana branch. In other words, lower fold-bias of genes with LSTs to testis-biased (or ovary-biased) expression than those with clade-wide biases would only be expected if the LSTs in fact occurred in Dana, rather than being reverse transitions in the shared ingroup branch. Together, it is evident that fold sex-biased gonadal expression has evolved dynamically in the Drosophila clade, and most importantly, there is evidence of a time-dependent increase in fold bias in both ovary- and testis-biased genes.

#### Multiple transitions in sex-biased status in Drosophila

It is worthwhile to mention that for our assessment of transitions in SBS above, we focused our main analysis on LSTs that had conserved SBS in three of four species branches, as these allowed us to assess the relative rate of different types of transitions. Whilst a majority of genes that exhibited variation in sex-biased expression across the Drosophila clade involved LSTs (*N* = 3,431 of 5331, 64.4%), a portion of genes exhibited multiple differences in SBS across the phylogeny (*N* = 1,900). Of these, 492 (25.9%) exhibited different SBS in in three of four lineages, and the remainder had two lineages with one SBS and the remaining two with another SBS (both paraphyletic and monophyletic cases). Specifically, 699 (36.7%) were unbiased (in two species) and ovary-biased (in the other two species), 683 (35.9%) were testis-based and unbiased genes, whilst only 26 (1.4%) were testis-biased and ovary-biased. These trends concur with results from LSTs (Table 2) where reversals appear comparatively uncommon. It is not possible to discern between the two types of reversals using this assessment, as was done for LSTs in Table 2, since the ancestral state cannot be determined for multiple transitions.

#### Genes without orthologs exhibit testis-biased expression

Our main analyses in the sections above were focused on all genes with one-to-one orthologs across all four Drosophila species. Nonetheless, it is useful to also consider the comparatively small subset of genes without orthologs. Of the 13,933 genes examined in the reference species Dmel, one-to-one orthologs were identified for 10,740 genes in the three sister species (Dsim, Dyak, and Dana; obtained from the Drosophila ortholog database at FlyBase) [49] that were used in our study, while the remaining 3,193 genes were excluded from our analysis. Of those excluded, 1,654 of these had more than one equal match in a species and were excluded for that reason (see Methods). The remaining 1,539 excluded Dmel genes lacked an ortholog match in at least one sister species.

Within the 1,539 genes without orthologs, 52.5, 14.3 and 33.1% were testis-biased, ovary-biased or unbiased respectively in Dmel (Chi^2^ *P* < 0.0001 for each paired contrast). Thus, a relatively high proportion of genes that lack between-species orthologs were testis-biased, concurring with patterns previously suggested for male-biased genes in Drosophila [8, 14]. Male-biased genes have often been observed to have fast protein sequence divergence (see the section for testis-biased genes studied here, “*Analyses of protein divergence of sex-biased gonadal genes*”) [1], which may act to prevent identification of orthologs, and/or can lead to gene losses from the genome. The trends are also consistent with data from gonads showing that de novo genes are preferentially involved in testis functions [29, 62]. In the genus Anopheles, fewer ortholog matches were observed for both strongly ovary- and testis-biased genes [23] again suggesting gonadal sex-biases may be associated with loss of orthologs. In sum, the subset of genes excluded from our main analyses are preferentially testis-biased and thus may have experienced too rapid divergence to yield identifiable orthologs, gene loss in some lineages, and/or arisen from de novo gene gains.

### Analyses of protein divergence of sex-biased gonadal genes

As a follow-up analysis, given that rapid protein sequence functional divergence (reflected by dN/dS) has often been associated with male-biased expression (as compared to female or unbiased) in Drosophila [1, 8, 10, 11, 15], we assessed how dN/dS varied among our various gene sets herein. In available studies of sex-biased expression in Drosophila, expression has often been determined in one single species (usually Dmel) and dN/dS measured using sequence data across various related species [6, 31]. Here, we have the fortunate advantage of having both sex-biased gonadal expression status, and genomic sequence data for all studied taxa, and thus can assess the relationship between sex-biased expression in the reproductive organs and dN/dS in each of the four species, including genes with LSTs and with clade-wide sex-biased status.

**Fig. 5:**
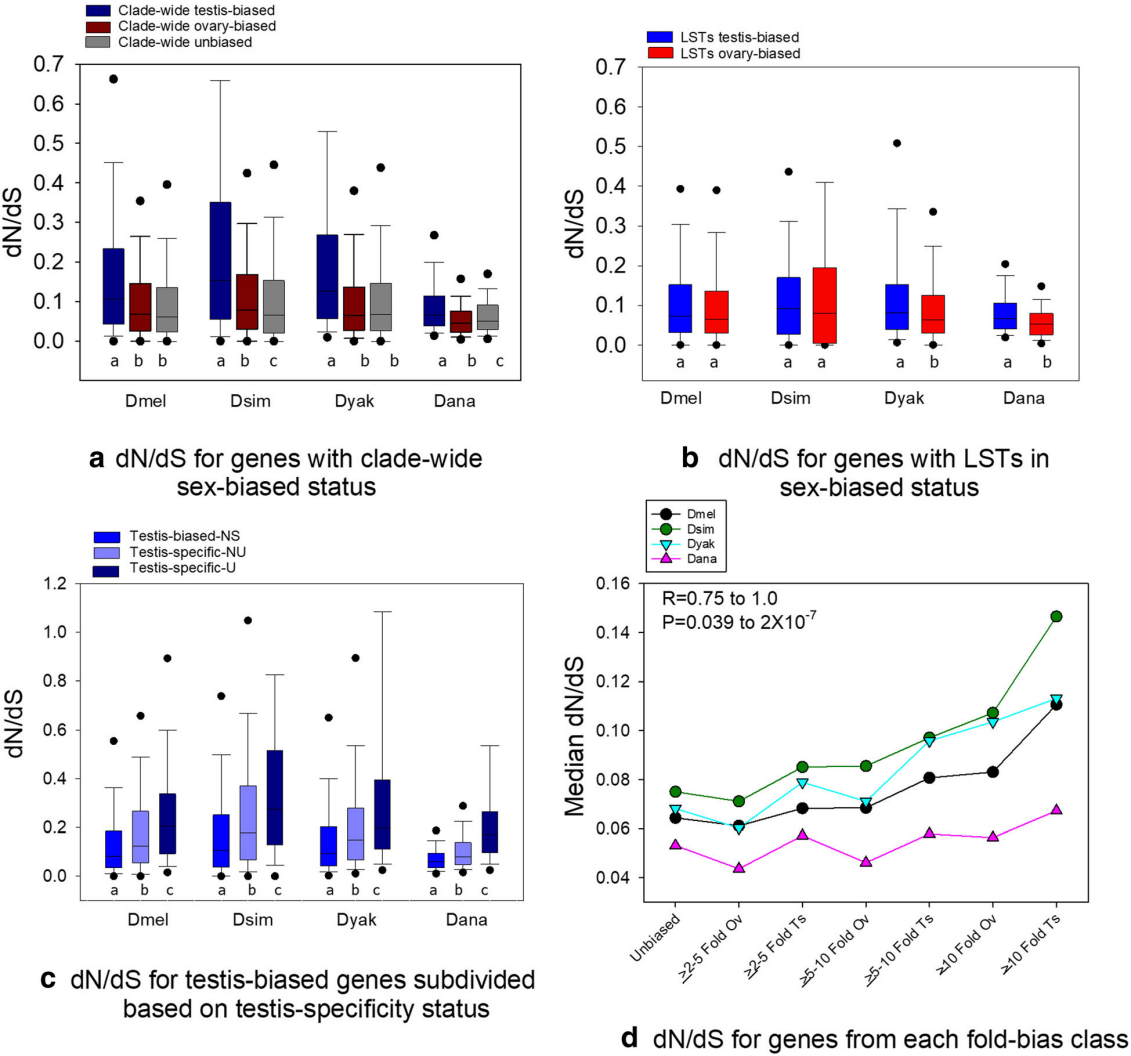
Summary of dN/dS values from various gene sets for the four species of Drosophila**. a**) Box plots of dN/dS values for genes with clade-wide testis-biased, ovary-biased and unbiased expression per species (clade-wide biases shown in dark red, blue, grey); **b**) Box plots of dN/dS values for the subset of genes with LSTs from unbiased to testis-biased status and unbiased to ovary-biased status (standard blue, red); **c**) dN/dS for testis-biased genes subdivided by testis-specificity, that includes genes that were testis-biased but not testis-specific (testis-biased-NS), genes that were testis-specific and not universally testis-specific (testis-specific-NU), and universally testis-specific genes (testis-specific-U); and **d**) The median dN/dS values for testis-biased and ovary-biased genes per fold bias category and unbiased genes for each species under study. Different letters under each set of grouped bars in panel **a** and **c** indicate a statistically significant difference (ranked ANOVA and Dunn’s paired contrast (P < 0.05) and between the two species per group in panel **b** (MWU-tests P < 0.05). In panel **d**, Spearman’s R and *P* values for paired contrasts of species are shown. Ts = testis-biased, Ov = ovary-biased. Also, for each fold bias category per species in panel **d**, testis-biased genes had higher dN/dS than ovary-biased genes (MWU-test P < 0.05)

A summary of the results for dN/dS are presented in Fig. 5a-d. As shown in Fig. 5a, the genes with clade-wide testis-biased status had higher dN/dS than their ovary-biased and unbiased counterparts in each of the four species (Fig. 5a, ranked ANOVA and Dunn’s contrast *P* < 0.05 per species), similar to patterns observed in some Drosophila lineages [15]. Similar results were obtained across all sex-biased genes per species as shown in Additional file 1: Fig. S3. However, dN/dS of the testis-biased genes with clade-wide status (Fig. 5a) had higher dN/dS than all testis-biased genes per species (except Dana, MWU-test P < 0.05), and thus conserved testis-biased expression appears to be linked to accelerated protein sequence divergence as opposed to those with variability in testis-biased expression.

We compared dN/dS of genes with LSTs from unb-ts and unb-ov expression, which should indicate whether protein evolution was accelerated in testis-biased (as compared to ovary-biased) genes in the period following the transition from unbiased to sex-biased status in a single branch. As shown in Fig. 5b, we found that the median dN/dS was elevated for genes exhibiting unb-ts transitions as compared to unb-ov transitions for each of the four species under study. However, the difference was only statistically significant for the two basally branching species Dyak and Dana, which might reflect the reduced time period to accumulate amino acid changes in Dsim and Dmel. This could suggest that genes that have accumulated substitutions for shorter periods of time exhibit greater noise in dN/dS (Fig. 5b), reducing power to detect differences. For instance, it may be possible that regulatory changes may precede functional changes in some genes after a transition to sex-biased gonadal expression. Nonetheless, these findings suggest the initial emergence of faster evolution in testis-biased genes (than ovary-biased) can be weakly observed in as little as 5 My and is more evident with greater divergence time, suggesting the male-female effect may strengthen (not magnify, but lead to a more homogenous male and female dN/dS per species) over time.

As proteins of testis-biased genes consistently evolved faster than ovary-biased and unbiased genes herein (Fig. 5ab), we aimed to further study this group. For this, the testis-biased genes per species were divided into three distinct categories based on testis-specificity (defined herein as relative to ovary). The categories (per species) were: 1) testis-biased genes that were not testis specific (testis-biased-NS); 2) testis-specific genes that were not universally (clade-wide) testis-specific (testis-specific-NU), and 3) universally (clade-wide) testis-specific genes (testis-specific-U). The results show that dN/dS increased markedly from the first, to the second, to the third of these categories for each of the four species (Fig. 5c). In particular, for each species the testis-specific-U genes had statistically significantly higher dN/dS per species than testis-specific-NU and than testis-biased-NS (Fig. 5c). Thus, the longer testis-specific expression of a gene has been conserved within this clade, the higher the rate of protein evolution were for that gene. Too few genes had ovary-specific status for us to be able to perform a meaningful parallel analysis for those tissues (Table 1), consistent with fewer genes playing roles that are specific to the female gonad, to the exclusion of testis expression (functions of genes with clade-wide testis-specificity are shown in Table S9 and discussed in Additional file 1: Text File S3).

We next assessed the relationship of fold-sex biased expression and dN/dS in all four species under study. As shown in Fig. 5d, fold sex-bias in these four Drosophila species exhibited a remarkably consistent relationship with dN/dS. In particular, genes with ≥10-fold testis-biased expression had the highest median dN/dS values for each Drosophila species under study. The ≥10-fold class of testis-biased genes had higher dN/dS than testis-biased genes with lower fold testis-bias (between ≥2 to 10-fold bias), than all fold-bias classes of ovary-biased genes, and than unbiased genes (MWU-tests *P* < 0.05). In addition, dN/dS was higher for testis-biased than ovary-biased genes in all three of the fold-bias categories (MWU-test P < 0.05). The patterns observed for dN/dS across fold-biased categories was remarkably consistent across all species with Spearman’s R ≥ 0.75 (*P* ≤ 0.039) for each pairwise contrast between species (Fig. 5). Thus, fold sex-biased expression in the gonads is linked to dN/dS in each of these four species.

Protein sequence divergence data from the flyDIVaS database [59], which contains dN/dS values for six species from the melanogaster group (our four studied species, as well as *D. sechellia* and *D. erecta*) from the M0 model (M0 provides one dN/dS across all species) were compared to our M1 values (using mean dN/mean dS across four species branches) spanning all studied genes herein. We found a strong Spearman’s correlation (R = 0.86, *P* < 2X10^− 7^) between those genes common to both datasets (with orthologs, and unsaturated in each set). Thus, the results affirm congruence amongst dN/dS values between approaches.

#### Pleiotropy may shape dN/dS

**Fig. 6:**
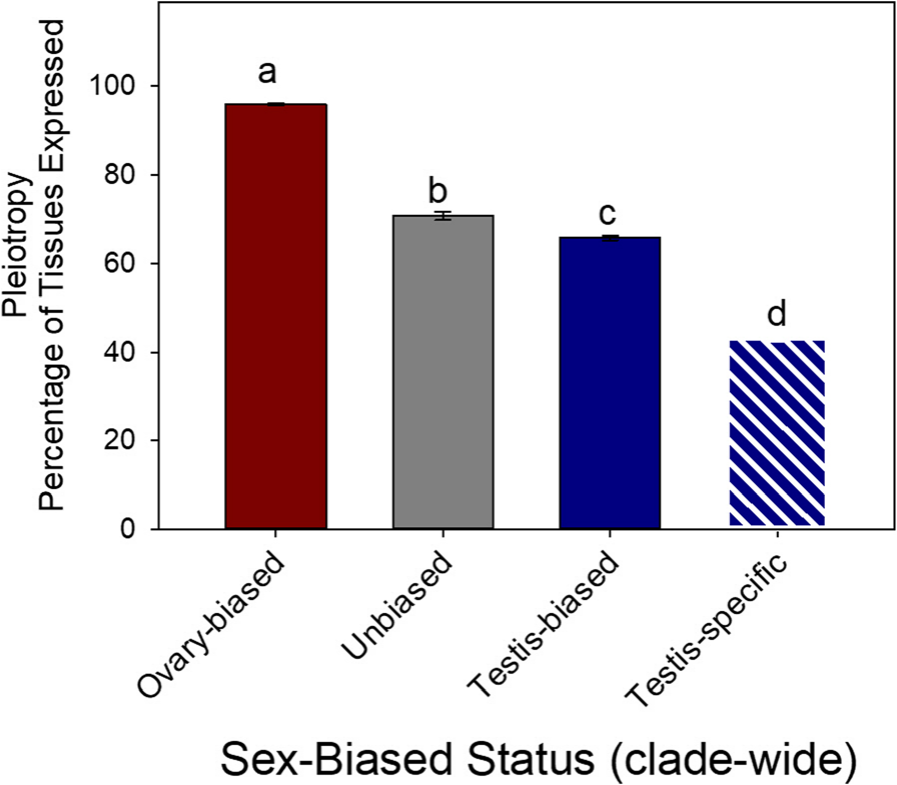
The expression breadth for genes with clade-wide (conserved) sex-biased status in all four species. Expression breadth per gene was determined as the percentage of 17 tissues with expression using data from *D. melanogaster*. Different letters above bars in indicate a statistically significant difference (P < 0.05) using ranked ANOVA and Dunn’s paired contrast. Note dark red, grey and blue are used here to denote clade-wide biases. Bars are average expression breadth per category, and error bars denote standard errors. Note that testis-specific expression status in the figure was defined based on comparison to the ovaries

To evaluate the role of pleiotropy on dN/dS herein, we assessed expression breadth of clade-wide (universally) testis-biased, universally ovary-biased and unbiased genes, as these gene sets had the same SBS in all species. As shown in Fig. 6, using Dmel as the reference for expression breadth, we found that universally ovary-biased genes exhibited very high expression breadth, with an average percent expression of 96.02% (standard error ± 0.29) across the various tissues/stages, while in contrast, universally unbiased genes had markedly lower expression breadth values of 70.72% (±0.95) (Ranked ANOVA P and Dunn’s contrast < 0.05). Clade-wide testis-biased genes had even lower expression breadth than comparable ovary- and unbiased genes, at only 65.71% (±0.54; MWU-test P < 0.05). Furthermore, exceptionally low expression breadth was observed for the clade-wide testis-specific genes, which exhibited on average only 43.1% (±0.88), less than half that found for ovary-biased genes, and more than 22 percentage points lower than unbiased genes or testis-biased genes. If we limit this analysis to Dmel, wherein both expression breadth and dN/dS were determined in the same species, we find higher dN/dS occurred in those gene sets with lower pleiotropy (e.g., testis-biased, testis-specific, Figs. 5ac, 6). Each of the three other species (Dsim, Dyak and Dana) also showed precisely the same patterns (Figs. 5ac, 6), thus adding support to this finding. Collectively, these data are consistent with the hypothesis that pleiotropy shapes protein sequence divergence [8, 14, 41]; testis-biased genes, and especially clade-wide testis-specific genes, may evolve rapidly in protein sequence due to low pleiotropy.

Further to the evidence that pleiotropy explains divergence in proteins with clade-wide sex-biased expression, we found only a slightly higher rate (< 3%, Chi^2^ *P* > 0.05) of positive selection amongst testis-biased as compared to ovary-biased and unbiased genes using sites analysis in PAML [57], which was not a statistically significant difference (Additional file 1: Text File S4, Table S10). Similar results were obtained using sites analysis of the melanogaster group of Drosophila that is available from flyDIVaS [59], which provides more powerful positive selection tests across six species from this group (Additional file 1: Text File S4, Table S10). In addition, we tested for positive selection in genes exhibiting LSTs from unbiased to sex-biased status, that is unb-ts and unb-ov (Table 2, Additional file 1: Table S11) using branch-site analyses [57] with the branch having the LST being tested for positive selection (see also Additional file 1: Text File S4). No significant differences were found in rates of positive selection, even in these genes with recent transitions to sex-biased status. These patterns further concur with a significant role of pleiotropy in shaping the protein sequence divergence of sex-biased genes in these species (Figs. 5ac, 6).

## Discussion

Drosophila has served as a primary model system for the study of sex-biased gene expression in metazoans. An array of studies have assessed the dynamics of sex-biased expression as well as its effects on protein sequence divergence [1, 2, 4, 6, 10, 14, 15, 29–38]. These studies often include gene expression in whole males versus females in Dmel, and sometimes involve a sister species and/or some sexual tissues. While multi-species studies of sex-biased gene expression in the Drosophila genus have been uncommon [8, 11], a seminal investigation of whole males and females was conducted by Zhang et al. [8]. The various findings therein included that species-restricted (orphan) genes tended to be male-biased in expression, changes in sex-biased expression (female:male expression ratio) accumulated monotonically over time, and that the between-species variation (standard deviation of female:male expression ratio) was higher in male-biased (than female-biased) genes [8], the latter patterns which are consistent with our results based on gonadal expression in Figs. 1b and 2b. That study, and an array of additional investigations in Drosophila, have also shown that male-biased genes, including reproductive genes, exhibit elevated dN/dS (than female-biased and/or unbiased), a trend consistent with observations in most, but not all [22, 23], metazoan models studied to date [1, 4, 6, 10, 11, 28, 30, 31, 35].

As sex biases mostly originate from the sex-organs [3, 6, 8, 22, 33], the specific study of gonads should lead to the most accurate picture of differences sex biases, unhampered by dilution of expression by the nongonadal somatic tissues [35, 39]. We thus conducted multi-species contrasts of sex-biased expression specifically from the sex-organs, and quantified each of six different types of interspecies transitions in sex-biased gonadal status (between ovary-biased, testis-biased and unbiased status) over different evolutionary time periods from (5, 13 and 44 My). We separately assessed expression across nongonadal tissues, using expression breadth. These approaches allowed us an original framework to consider the roles of selection and pleiotropy in the evolution of sex-biased gonadal gene expression.

### The present results in context

#### Fold sex-biased expression

Our present results add several elements to our current knowledge of the evolution of sex-biased expression in Drosophila. The results revealed a significant phenomenon, namely a directional stepwise increase in the degree of sex-biased gonadal expression with extended periods of conserved sex-biased status (Fig. 4). Note that this pattern is not between species variation (or standard deviation) in the female:male expression ratio as was reported in male-female contrasts [8], nor is it a decreased correlation in FPKM of gonadal genes between species over time (My) as was shown in Fig. 2b. Remarkably, the progressive increase in fold-sex-bias was separately observed for testis-biased and for ovary-biased genes in this genus (Fig. 4). We speculate that the directional increase in fold sex-bias might be explained by a model of episodic inter-locus sexual antagonism. In other words, this may reflect male and female reactionary responses in gene expression evolution. Inter-locus sexual conflict has been thought to possibly underlie sex-biases in expression, particularly in polyandrous species such as in Drosophila [1, 27]. In this aspect, our findings may be deemed consistent with earlier proposals from analyses in the species Dmel, which have suggested that a portion of sex-biased genes and gene networks may evolve under inter-locus sexual antagonism in that organism [38, 63]. While some lability of sexual dimorphism, and thus expression, might sometimes be experienced in closely related species such as Dmel and Dsim (< 5 My) [64], that would not be expected to be directional, nor to occur across 44 My as observed herein (Fig. 4). We contemplate that if sexual antagonism is behind the patterns observed here for fold bias of testis- and ovary-biased genes, it need not necessarily have been ongoing continuously for 44 My in this genus, but may instead have arisen in episodic bursts in each branch (cf. [65]). Together, it may be proposed that selective processes, and potentially sexual antagonism, contribute to this phenomenon.

Nevertheless, the current understanding of the relationship between sexual antagonism and sex-biased gene expression, particularly inter-locus sexual conflict, remains at the early stages [16, 18, 19, 27]. Thus, while the interpretation of these results remains speculative, future research on genes with enhanced fold sex-bias over time could provide a valuable avenue for further study of inter-locus sexual antagonisms in this genus.

#### Selection and frequency of S-LSTs

We found evidence that the six types of LSTs in gonadal SBS, after standardization to the number of genes with the ancestral state (S-LSTs), occurred at markedly different rates in this genus (Table 2). This pattern was observed for each of four species branches studied, and suggests differential selection has at least partly contributed towards shaping transitions in sex-biased status in the gonads. This finding concurs with prior reports suggesting that reversals are generally uncommon, primarily from whole males/females [3, 8, 11, 14]. The data specifically from the gonads that we analysed herein further refine that pattern, and indicates that the two types of reversals may exhibit differences in rates of turnover, with remarkably few ov-ts transitions (as compared to ts-ov) in the more recently diverged branches Dmel and Dsim (5 My). In this regard, we speculate that a transition from ovary-biased status to testis-biased status may be particularly detrimental, putatively affecting fitness in a more deleterious manner than the reverse type of transition. This could reflect the fact that female sexual organs play multiple crucial roles in fitness, not only by forming and protecting the egg, but also by housing and maintaining the sperm in the spermathecae and seminal receptacle (wherein sperm are stored after mating). The ovaries, and the process of ovulation, may be involved in the transfer of sperm from the reproductive tract into the spermathecae, and in the later release of sperm from the spermathecae for sperm-egg fertilization [22, 66] . Thus, given such essential fitness roles, one can contemplate that a drastic change (a reversal from ovary-biased status to testis-biased status) in sex-biased expression in ovaries (ov-ts) might typically be more deleterious than for the testes (ts-ov), potentially leading to lower levels of S-LSTs.

It has been suggested that in an ant (*Solenopsis invicta*), new forms of biased gene expression may be facilitated by relaxed selection, which allows flexibility for alternate phenotypes [67]. In this regard, one possibility is that gains/losses in sex-biased gonadal status within a species branch might resolve the ongoing need for variation (at intermittent periods) in reproductive traits or strategies in Drosophila during its evolution, accelerating turnover. In contrast, reversals, particularly ov-ts reversals, may be too deleterious for such variation, and thus have been subjected to high purifying selection.

Theory has predicted that sexual selection acts on sex-biased gene expression [1], and recent experimental research had provided support for this concept. For instance, in Dmel it was shown that when flies were forced into monogamy (versus the natural state of polyandry), there was greater expression of female-biased genes and reduced transcription of male-biased genes [68]. Those findings are consistent with the notion that male-biased expression is influenced by sexual selection, such as female choice or male-male competition [1, 68, 69]. An additional study using gonadal expression data from birds has shown that sexual selection in males (e.g. ornamentation) is correlated to the turnover rate of testis-biased expression [19]. It has also been hypothesized that sex-biased gene expression evolves adaptively in Drosophila [32]. In this regard, some of the variation in Table 2 might result not only from differences the degree of purifying selection, but also from adaptive changes.

Moreover, in terms of clade-wide conserved sex-biased status, we found an absence of clade-wide ovary-specific genes (defined by expression contrasts to testis) as compared to testis-specific genes, which were relatively common (Table 1, Table S3). This suggests greater sexual specialization of male gonadal transcription. This clade-wide testis-specificity may have been mediated by sex-related purifying and/or positive selection over 44 My in this taxon, and appears inconsistent with a purely neutral model. Putative olfactory roles of genes that transitioned from unb-ts in all four species (Additional file 1: Tables S6, S7) suggests that new or expanded roles of olfactory genes may play a significant role in interspecies gonadal divergence.

#### Pleiotropy

Importantly, our approach of studying S-LSTs in SBS in Drosophila allowed an original and rare empirical test of the fundamental hypothesis that pleiotropy restricts the evolution of sex-biased expression in metazoans [25]. By comparing S-LSTs to expression breadth we could assess whether changes in sex-biased status were restricted by cross-tissue pleiotropy in Dmel (Fig. 3). While it has been postulated that genes with functions in multiple tissues and processes act to impede interspecies evolution of sex-biased expression [25–27, 41], and that low pleiotropy would facilitate shifts towards sex-biased expression, we did not observe a consistent connection between pleiotropy and the rate of transitions in SBS in the Dmel gonads studied here (Fig. 3ab). It should be noted nonetheless that clade-wide testis-biased genes did exhibit lower pleiotropy than ovary-biased genes (Fig. 6), and testis expression (FPKM) as a whole (across all genes) diverged more rapidly over time (than ovary, Fig. 2b). However, the frequency of each type of transition in SBS were largely unrelated to gene pleiotropy (Fig. 3ab). Further study, potentially using similar approaches as those employed herein in a broader range of organisms, may help further decipher the role of pleiotropy in evolution of sex-biased gonadal expression patterns.

While pleiotropy appeared largely unlinked to the rate of transitions in SBS (Fig. 3), we did find evidence that low pleiotropy may explain high dN/dS of testis-biased genes, and particularly clade-wide testis specific genes (Figs. 5, 6; Additional file 1: Text File S4). Thus, the results add to the growing support for the hypothesis that low pleiotropy allows functional divergence of the proteins encoded by sex-biased genes [8, 14, 41]. There is at least one feasible explanation for the different roles of pleiotropy in evolution of sex-biased expression (no detectable effect), and in shaping dN/dS. Specifically, a change in the protein sequence of an ovary- or testis-biased gene that is broadly expressed in sexual and non-sexual tissues, would likely affect its phenotypes in the non-sexual organs [41]. In contrast, a change in the gonadal SBS of a gene could primarily or solely affect roles in the sexual organs, and not necessarily affect its functions or genetic pathways in other nongonadal tissues. In this context, transitions in sex-biased gonadal expression, unlike dN/dS, could often be independent of pleiotropy.

#### Patterns observed for dN/dS across species

It is worthwhile to consider that our findings of higher dN/dS for testis-biased genes than for ovary-biased genes in all four Drosophila species (Fig. 5abd, Additional file 1: Figure S3) concurs with patterns observed for male-biased genes in this genus [1, 11, 14, 15, 31]. The findings of higher dN/dS for testis-biased genes than for ovary-biased genes in Drosophila (Fig. 5abd, Additional file 1: Figure S3), differs markedly from results in fellow dipteran genera Aedes and Anopheles. In those mosquitoes, higher dN/dS occurred in ovary-biased (and ovary-specific) genes than their male counterparts [22, 23]. One key factor that could explain this difference is the effectiveness of post-mating mating plugs; which greatly impedes sperm competition in mosquitoes but not in Drosophila [22, 70, 71]. We speculate that this may lead to greater sperm competition and a propensity for adaptive evolution and higher dN/dS of testis-biased (than ovary-biased) in flies than in mosquitoes. However, elevated positive selection in testis-biased genes was only weakly observed herein (Additional file 1: Text File S4). Alternatively, low pleiotropy, as found here for testis-biased genes in flies (Fig. 6; see also [10, 31]) could occur for ovary-biased genes in mosquitoes, and thus this comprises a candidate hypothesis to explain these inter-taxon differences.

In Fig. 5d, some variation in dN/dS was observed between species, with the consistently highest values observed in Dsim, intermediate values for Dmel and Dyak, and the lowest values for Dana (Fig. 5d), suggesting species-specific effects on the evolutionary rates of sex-biased genes. Such variation might reflect fundamental differences between taxa, for example, effective population size. When a population size is small, this may lead to greater fixation of slightly deleterious mutations via genetic drift, causing higher dN and thus higher dN/dS [72–74]. Accordingly, a hypothetical history of relatively smaller effective population sizes in Dsim than in the other species studied here, may accelerate dN/dS, whilst if Dana had larger historical population sizes, this could enhance the efficiency of purifying selection, reducing dN/dS. The effective population size of Drosophila’s various species, however, has been a subject of debate [73–76], such that further data will be needed to assess precisely whether and how it may be connected to dN/dS.

Alternatively, the interspecies variation may also reflect differences in the sexual traits of these taxa. As an example, sperm size is extremely variable in the Drosophila genus and may evolve adaptively due to post copulation female-sperm choice; sperm competition is also common in this taxon [77, 78]. Thus, differences in sexual selection pressures might partly contribute towards interspecies variation in dN/dS.

Crucially, the present findings also showed that differences in dN/dS for genes with testis-biased and ovary-biased were more readily detected after 13 and 44 My than for genes with recently acquired SBS (5 My, Fig. 5b). This comprises a significant cautionary note for future studies of sex-biased genes in metazoans. That is, when studying sex-biased gene expression in only one or two species, one should be aware that the different genes under study may have experienced sex-biased expression for longer or shorter time periods (in one or two species studies this would be undetectable), which may affect observed levels of protein sequence divergence. Furthermore, our results showed that the observed fold-sex-biased expression (Fig. 4) is also time-dependent (which would also be undetectable in studies of one or two species). In this regard, future studies should consider the possibility that the ultimate dN/dS and fold-bias observed when assessing expression in one, or two, species may be the product of the largely hidden, or unknown, time-scales of conserved sex-biased expression.

#### Noteworthy caveats

It should be emphasized that measures of sex-biased expression in Drosophila have varied extensively in the literature, with estimates of the percent of sex-biased genes in the genome ranging between 10 and 91%, depending on statistical methods employed, the cutoff for identification of sex-biased status, size of transcriptome datasets and/or replication, and other factors including growth conditions [14]. As all species herein were from the same growth environments, and were subjected to the same statistical processes for all four species, the data are comparable to each other, and thus are aimed to reveal the relative variation in sex-biased gonadal expression in this genus using the criteria defined throughout our study. Thus, the patterns may vary to some extent with additional datasets, different criteria for defining sex-biased genes, or environmental conditions (for further consideration of approaches, including those used herein, see Additional file 1: Text File S5). Further studies in more taxa, and using various methods to assess sex-biased expression, will help discern the robustness of these patterns in Drosophila and across other organisms.

## Conclusions

While Drosophila has served as a core model system for investigation of sex-biased expression to date, our study shows that its utility for exploring factors shaping sex-biased evolution has not been exhausted. In the future, as more population level genomic sequence data sequence becomes available in various Drosophila species, research should aim to determine the relative selective pressures acting on mutations in sex-biased expression in populations using their frequency spectra, similar to that conducted for codon or protein mutations [74, 79], and to assess intra- versus interspecies expression divergence profiles for various organisms [2, 32, 42]. Such studies will help fruther ascertain the relative roles of neutral evolution, purifying selection and adaptive changes on the evolution of sex-biased gonadal expression. In addition, studies of sex-biased expression in specific nongonadal organs/tissues such as those listed in Additional file 1: Table S2 may be useful in discerning whether any particular tissues share parallel, or opposite, biases to those found in the gonads in Drosophila. Such data could reveal sex-related networks relevant to both gonadal and nongonadal tissues, which may be valuable to understanding evolution of gonadal sex-biased expression. Further studies in this genus outside the melanogaster group, such as in the model Hawai’ian clade [80], may provide a particularly effective route for future assessments of the factors shaping evolution sex-biased gonadal expression.

## Additional file

Additional file 1: The file contains the supplementary Tables, Figures and Text Files. (PDF 667 kb)

## References

[1] Ellegren H, Parsch J (2007). The evolution of sex-biased genes and sex-biased gene expression. Nat Rev Genet.

[2] Meiklejohn CD, Parsch J, Ranz JM, Hartl DL (2003). Rapid evolution of male-biased gene expression in Drosophila. Proc Natl Acad Sci U S A.

[3] Ranz JM, Castillo-Davis CI, Meiklejohn CD, Hartl DL (2003). Sex-dependent gene expression and evolution of the Drosophila transcriptome. Science.

[4] Zhang Z, Hambuch TM, Parsch J (2004). Molecular evolution of sex-biased genes in Drosophila. Mol Biol Evol.

[5] Cutter AD, Ward S (2005). Sexual and temporal dynamics of molecular evolution in C. Elegans development. Mol Biol Evol.

[6] Proschel M, Zhang Z, Parsch J (2006). Widespread adaptive evolution of Drosophila genes with sex-biased expression. Genetics.

[7] Yang X, Schadt EE, Wang S, Wang H, Arnold AP, Ingram-Drake L, Drake TA, Lusis AJ (2006). Tissue-specific expression and regulation of sexually dimorphic genes in mice. Genome Res.

[8] Zhang Y, Sturgill D, Parisi M, Kumar S, Oliver B (2007). Constraint and turnover in sex-biased gene expression in the genus Drosophila. Nature.

[9] Whittle CA, Malik MR, Krochko JE (2007). Gender-specific selection on codon usage in plant genomes. BMC Genomics.

[10] Haerty W, Jagadeeshan S, Kulathinal RJ, Wong A, Ram KR, Sirot LK, Levesque L, Artieri CG, Wolfner MF, Civetta A (2007). Evolution in the fast lane: rapidly evolving sex-related genes in Drosophila. Genetics.

[11] Jiang ZF, Machado CA (2009). Evolution of sex-dependent gene expression in three recently diverged species of Drosophila. Genetics.

[12] Small CM, Carney GE, Mo Q, Vannucci M, Jones AG (2009). A microarray analysis of sex- and gonad-biased gene expression in the zebrafish: evidence for masculinization of the transcriptome. BMC Genomics.

[13] Ometto L, Shoemaker D, Ross KG, Keller L (2010). Evolution of gene expression in fire ants: the effects of developmental stage, caste, and species. Mol Biol Evol.

[14] Assis R, Zhou Q, Bachtrog D (2012). Sex-biased transcriptome evolution in Drosophila. Genome Biol Evol.

[15] Grath S, Parsch J (2012). Rate of amino acid substitution is influenced by the degree and conservation of male-biased transcription over 50 myr of Drosophila evolution. Genome Biology and Evolution.

[16] Parsch J, Ellegren H (2013). The evolutionary causes and consequences of sex-biased gene expression. Nat Rev Genet.

[17] Whittle CA, Johannesson H (2013). Evolutionary dynamics of sex-biased genes in a hermaphrodite fungus. Mol Biol Evol.

[18] Ingleby FC, Flis I, Morrow EH (2014). Sex-biased gene expression and sexual conflict throughout development. Cold Spring Harb Perspect Biol.

[19] Harrison PW, Wright AE, Zimmer F, Dean R, Montgomery SH, Pointer MA, Mank JE (2015). Sexual selection drives evolution and rapid turnover of male gene expression. Proc Natl Acad Sci U S A.

[20] Lipinska A, Cormier A, Luthringer R, Peters AF, Corre E, Gachon CM, Cock JM, Coelho SM (2015). Sexual dimorphism and the evolution of sex-biased gene expression in the brown alga ectocarpus. Mol Biol Evol.

[21] Wang X, Werren JH, Clark AG (2015). Genetic and epigenetic architecture of sex-biased expression in the jewel wasps Nasonia vitripennis and giraulti. Proc Natl Acad Sci U S A.

[22] Whittle CA, Extavour CG (2017). Rapid evolution of ovarian-biased genes in the yellow fever mosquito (Aedes aegypti). Genetics.

[23] Papa F, Windbichler N, Waterhouse RM, Cagnetti A, D’Amato R, Persampieri T, Lawniczak MK, Nolan T, Papathanos PA (2017). Rapid evolution of female-biased genes among four species of Anopheles malaria mosquitoes. Genome Res.

[24] Congrains C, Campanini EB, Torres FR, Rezende VB, Nakamura AM, JLd O, Lima ALA, Chahad-Ehlers S, Sobrinho IS, RAd B (2018). Evidence of adaptive evolution and relaxed constraints in sex-biased genes of south American and West Indies fruit flies (Diptera: Tephritidae). Genome Biology and Evolution.

[25] Mank JE, Hultin-Rosenberg L, Zwahlen M, Ellegren H (2008). Pleiotropic constraint hampers the resolution of sexual antagonism in vertebrate gene expression. Am Nat.

[26] Dean R, Mank JE (2016). Tissue specificity and sex-specific regulatory variation permit the evolution of sex-biased gene expression. Am Nat.

[27] Mank JE, Wedell N, Hosken DJ (2013). Polyandry and sex-specific gene expression. Philos Trans R Soc Lond Ser B Biol Sci.

[28] Grath S, Parsch J (2016). Sex-biased gene expression. Annu Rev Genet.

[29] Begun DJ, Lindfors HA, Kern AD, Jones CD (2007). Evidence for de novo evolution of testis-expressed genes in the Drosophila yakuba/Drosophila erecta clade. Genetics.

[30] Jagadeeshan S, Singh RS (2005). Rapidly evolving genes of Drosophila: differing levels of selective pressure in testis, ovary, and head tissues between sibling species. Mol Biol Evol.

[31] Meisel RP (2011). Towards a more nuanced understanding of the relationship between sex-biased gene expression and rates of protein-coding sequence evolution. Mol Biol Evol.

[32] Nuzhdin SV, Wayne ML, Harmon KL, McIntyre LM (2004). Common pattern of evolution of gene expression level and protein sequence in Drosophila. Mol Biol Evol.

[33] Parisi M, Nuttall R, Edwards P, Minor J, Naiman D, Lü J, Doctolero M, Vainer M, Chan C, Malley J (2004). A survey of ovary-, testis-, and soma-biased gene expression in Drosophila melanogaster adults. Genome Biol.

[34] Parisi M, Nuttall R, Naiman D, Bouffard G, Malley J, Andrews J, Eastman S, Oliver B (2003). Paucity of genes on the Drosophila X chromosome showing male-biased expression. Science.

[35] Perry JC, Harrison PW, Mank JE. The Ontogeny and Evolution of Sex-Biased Gene Expression in *Drosophila melanogaster*. 2015;31(5):1206–19. 10.1093/molbev/msu072 PMC3995337 24526011

[36] Arbeitman MN, Furlong EE, Imam F, Johnson E, Null BH, Baker BS, Krasnow MA, Scott MP, Davis RW, White KP (2002). Gene expression during the life cycle of Drosophila melanogaster. Science.

[37] Churchill GA, Oliver B (2001). Sex, flies and microarrays. Nat Genet.

[38] Hansen ME, Kulathinal RJ (2013). Sex-biased networks and nodes of sexually antagonistic conflict in Drosophila. Int J Evol Biol.

[39] Chintapalli VR, Wang J, Dow JA (2007). Using FlyAtlas to identify better *Drosophila melanogaster* models of human disease. Nat Genet.

[40] Böhne A, Sengstag T, Salzburger W (2014). Comparative transcriptomics in east African cichlids reveals sex- and species-specific expression and new candidates for sex differentiation in fishes. Genome Biology and Evolution.

[41] Mank JE, Ellegren H (2009). Are sex-biased genes more dispensable?. Biol Lett.

[42] Khaitovich P, Hellmann I, Enard W, Nowick K, Leinweber M, Franz H, Weiss G, Lachmann M, Pääbo S (2005). Parallel patterns of evolution in the genomes and transcriptomes of humans and chimpanzees. Science.

[43] Dorus S, Busby SA, Gerike U, Shabanowitz J, Hunt DF, Karr TL (2006). Genomic and functional evolution of the *Drosophila melanogaster* sperm proteome. Nat Genet.

[44] Boes KE, Ribeiro JM, Wong A, Harrington LC, Wolfner MF, Sirot LK (2014). Identification and characterization of seminal fluid proteins in the Asian tiger mosquito, *Aedes albopictus*. PLoS Negl Trop Dis.

[45] Torgerson DG, Kulathinal RJ, Singh RS. Mammalian sperm proteins are rapidly evolving: evidence for positive selection in functionally diverse genes. Mol Biol Evol. 2002;2002, 19. 10.1093/oxfordjournals.molbev.a004021 12411606

[46] Swanson WJ, Vacquier VD (2002). The rapid evolution of reproductive proteins. Nat Rev Genet.

[47] Oliver TA, Garfield DA, Manier MK, Haygood R, Wray GA, Palumbi SR (2010). Whole-genome positive selection and habitat-driven evolution in a shallow and a deep-sea urchin. Genome Biol Evol.

[48] Vicens A, Lüke L, Roldan ER (2014). Proteins involved in motility and sperm-egg interaction evolve more rapidly in mouse spermatozoa. PLoS One.

[49] Gramates LS, Marygold SJ, Santos GD, Urbano JM, Antonazzo G, Matthews BB, Rey AJ, Tabone CJ, Crosby MA, Emmert DB, et al. FlyBase at 25: looking to the future. Nucleic Acids Res. 2016. 10.1093/nar/gkw1016 PMC5210523 27799470

[50] Rogers RL, Shao L, Sanjak JS, Andolfatto P, Thornton KR (2014). Revised annotations, sex-biased expression, and lineage-specific genes in the Drosophila melanogaster group. G3 (Bethesda).

[51] Kearse M, Moir R, Wilson A, Stones-Havas S, Cheung M, Sturrock S, Buxton S, Cooper A, Markowitz S, Duran C (2012). Genious basic: an integrated and extendable desktop software platform for the organization and analyiss of sequence data. Bioinformatics.

[52] Eisen MB, Spellman PT, Brown PO, Botstein D (1998). Cluster analysis and display of genome-wide expression patterns. Proc Natl Acad Sci U S A.

[53] Mank JE, Hultin-Rosenberg L, Axelsson E, Ellegren H (2007). Rapid evolution of female-biased, but not male-biased, genes expressed in the avian brain. Mol Biol Evol.

[54] Graveley BR, Brooks AN, Carlson JW, Duff MO, Landolin JM, Yang L, Artieri CG, van Baren MJ, Boley N, Booth BW (2011). The developmental transcriptome of Drosophila melanogaster. Nature.

[55] Tamura K, Subramanian S, Kumar S (2004). Temporal patterns of fruit fly (*Drosophila*) evolution revealed by mutation clocks. Mol Biol Evol.

[56] Kumar S, Stecher G, Peterson D, Tamura K (2012). MEGA-CC: computing core of molecular evolutionary genetics analysis program for automated and iterative data analysis. Bioinformatics.

[57] Yang Z (2007). PAML 4: phylogenetic analysis by maximum likelihood. Mol Biol Evol.

[58] Castillo-Davis CI, Bedford TB, Hartl DL (2004). Accelerated rates of intron gain/loss and protein evolution in duplicate genes in human and mouse malaria parasites. Mol Biol Evol.

[59] Stanley CE, Kulathinal RJ (2016). flyDIVaS: a comparative genomics resource for Drosophila divergence and selection. G3 (Bethesda).

[60] Huang d W, Sherman BT, Lempicki RA (2009). Systematic and integrative analysis of large gene lists using DAVID bioinformatics resources. Nat Protoc.

[61] Kang N, Koo J (2012). Olfactory receptors in non-chemosensory tissues. BMB Rep.

[62] Tautz D, Neme R, Domazet-Loso T. Evolutionary origin of orphan genes. eLS. 2013. 10.1038/nrg3053 21878963

[63] Innocenti P, Morrow EH (2010). The sexually antagonistic genes of Drosophila melanogaster. PLoS Biol.

[64] Capy PGP (2004). Drosophila melanogaster, Drosophila simulans: so similar yet so different. Genetica.

[65] Brawand D, Soumillon M, Necsulea A, Julien P, Csárdi G, Harrigan P, Weier M, Liechti A, Aximu-Petri A, Kircher M (2011). The evolution of gene expression levels in mammalian organs. Nature.

[66] Bloch Qazi MC, Heifetz Y, Wolfner MF (2003). The developments between gametogenesis and fertilization: ovulation and female sperm storage in Drosophila melanogaster. Dev Biol.

[67] Hunt BG, Ometto L, Wurm Y, Shoemaker D, Yi SV, Keller L, Goodisman MA (2011). Relaxed selection is a precursor to the evolution of phenotypic plasticity. Proc Natl Acad Sci U S A.

[68] Hollis B, Houle D, Yan Z, Kawecki TJ, Keller L (2014). Evolution under monogamy feminizes gene expression in *Drosophila melanogaster*. Nat Commun.

[69] Veltsos P, Fang Y, Cossins AR, Snook RR, Ritchie MG (2017). Mating system manipulation and the evolution of sex-biased gene expression in Drosophila. Nat Commun.

[70] Avila FW, Wong A, Sitnik JL, Wolfner MF (2015). Don't pull the plug! The Drosophila mating plug preserves fertility. Fly (Austin).

[71] Price CSC, Dyer KA, Coyne JA (1999). Sperm competition between *Drosophila* males involves both displacement and incapacitation. Nature.

[72] Kimura M (1983). The neutral theory of molecular evolution.

[73] Petit N, Barbadilla A (2009). Selection efficiency and effective population size in Drosophila species. Journal of Evolutioary Biology.

[74] Akashi H (1996). Molecular evolution between *Drosophila melanogaster* and *D. simulans*: reduced codon bias, faster rates of amino acid substitution, and larger proteins in *D. melanogaster*. Genetics.

[75] Vicario S, Moriyama EN, Powell JR (2007). Codon usage in twelve species of Drosophila. BMC Evol Biol.

[76] Nolte V, Schlotterer C (2008). African *Drosophila melanogaster* and *D. simulans* populations have similar levels of sequence variability, suggesting comparable effective population sizes. Genetics.

[77] Pitnick S (1996). Investment in Testes and the cost of making long sperm in Drosophila. Am Nat.

[78] Hosken DJ (2003). Dispatch. Sperm biology: size indeed matters. Curr Biol.

[79] Whittle CA, Sun Y, Johannesson H (2012). Genome-wide selection on codon usage at the population level in the fungal model organism *Neurospora crassa*. Mol Biol Evol.

[80] O'Grady P, DeSalle R (2018). Hawaiian Drosophila as an evolutionary model clade: days of future past. Bioessays.

