# Supplementary material for: Selection shapes turnover and magnitude of sex-biased expression in Drosophila gonads
Source: BMC Evol Biol. 2019 Feb 20;19:60. doi: 10.1186/s12862-019-1377-4 (PMC6383255; doi:10.1186/s12862-019-1377-4)
Supplement: Supplementary file 1 — The file contains the supplementary Tables, Figures and Text Files. (PDF 667 kb) [file 12862_2019_1377_MOESM1_ESM.pdf]

Additional File 1: Supplementary Figures, Tables and Text

to accompany

"Selection shapes turnover and magnitude of sex-biased expression in Drosophila gonads"

C.A. Whittle and C.G. Extavour

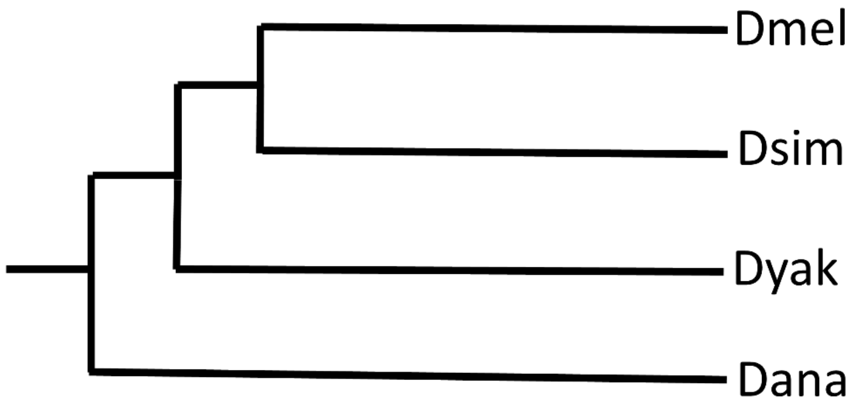

Fig. S1. The phylogeny of the four Drosophila species under study. Obtained from www.flybase.org.

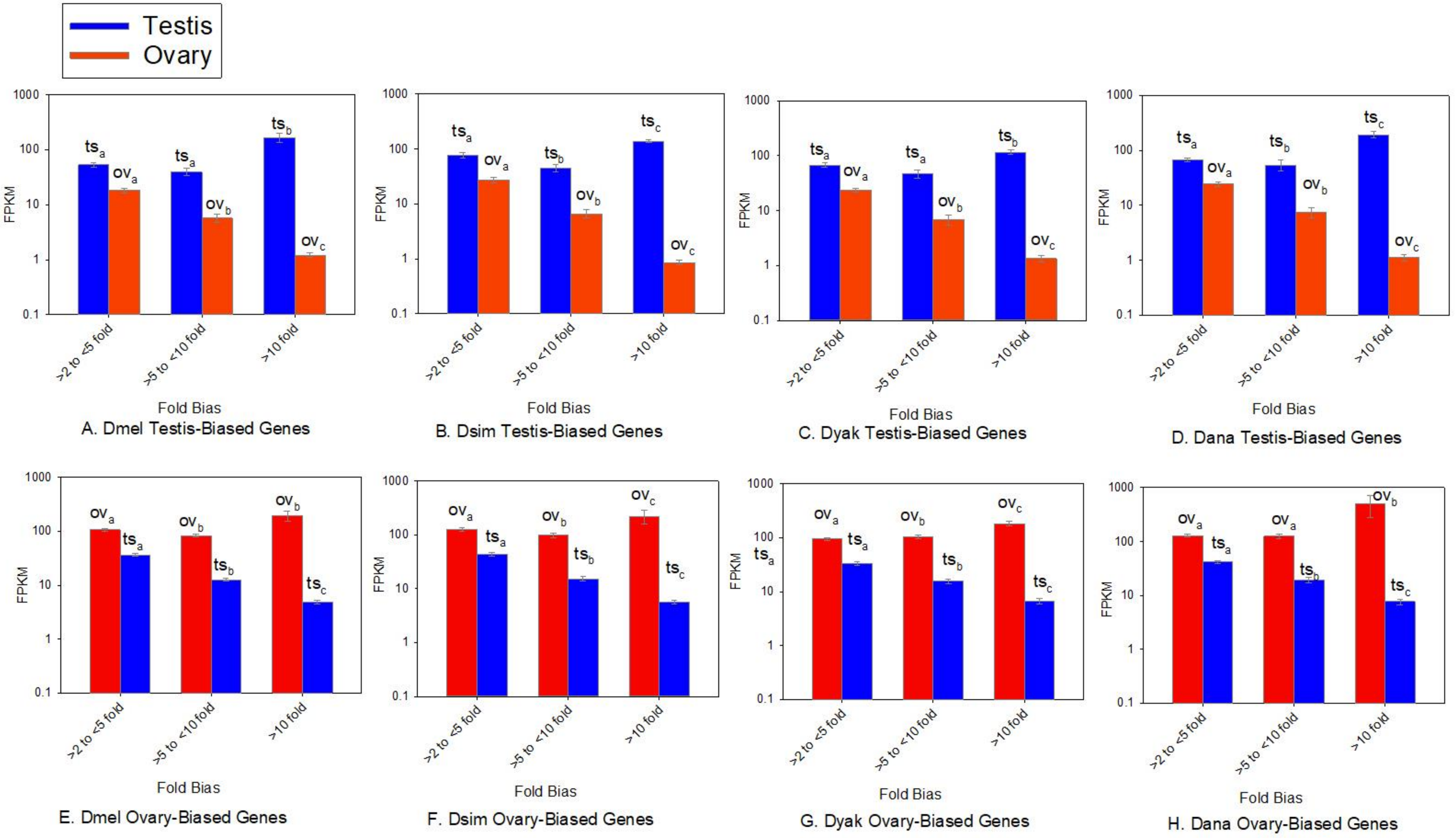

Fig. S2. Expression level of sex-biased gonadal genes. The average expression level (FPKM) level in the testes (ts) and ovaries (ov) with respect to fold-bias for testis-biased genes (A-D) and for ovary-biased genes (E-H) in all four species of Drosophila. Different subscript letters among ts and among ov bars in each Fig. indicate a statistically significant difference using a ranked ANOVA followed by Dunn's paired contrast (P<0.05). Error bards are standard errors.

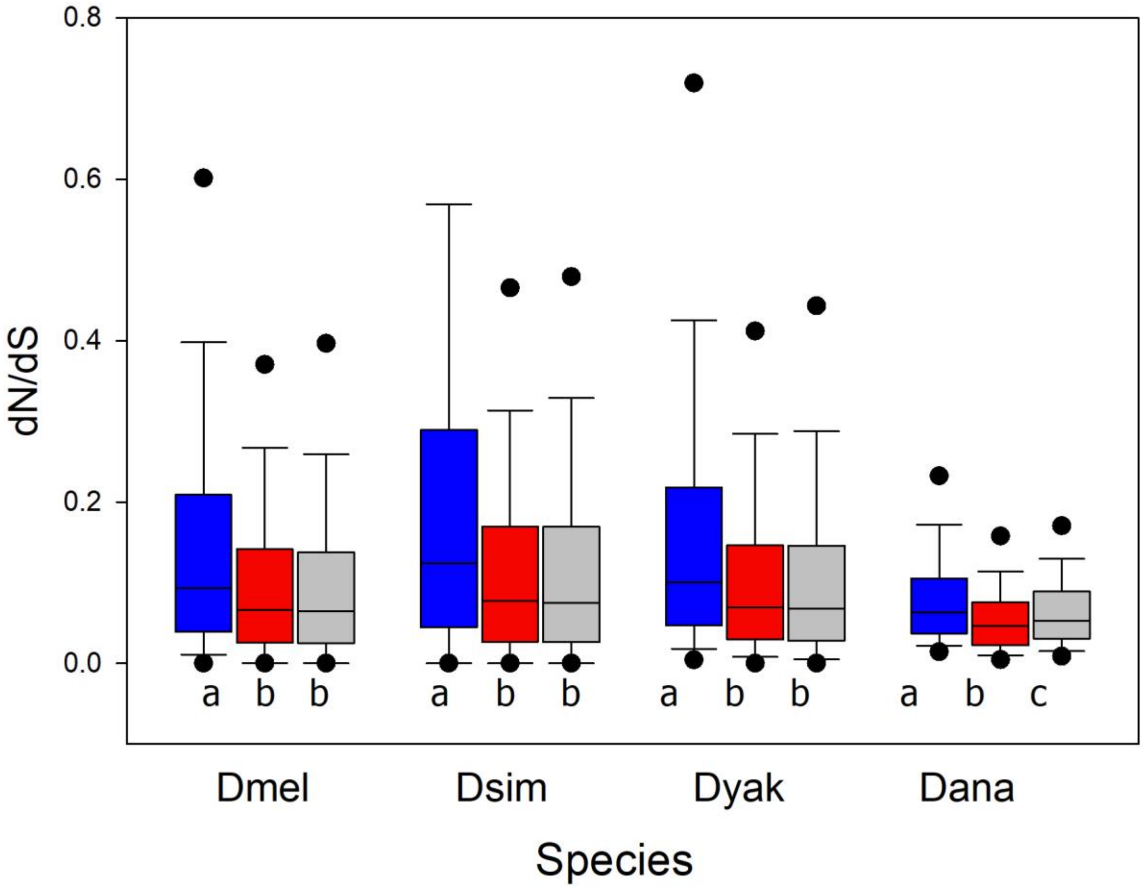

Fig. S3. Box plots of dN/dS for all testis-biased, ovary-biased and unbiased genes per species. Different letters below bars for each species indicate a statistically significant difference using Ranked ANOVA with Dunn's paired contrast ($P<0.05$). Blue=testis-biased, red=ovary-biased, grey=unbiased.

Table S1. The datasets under study in the present investigation and their location. All RNA-seq are from virgin males or females from a single experiment and grown under the same conditions (paired reads). See [1].

**Species**	**CDS at FlyBase (No. genes)**	**Tissue Studied for Expression**	**SRA Run ID**	**No. Spots**	**Dataset Description at SRA**[a]
D. ananassae	r1.050 (N=14,385)	Virgin male testis	SRR2021004	51,443,655	*D. ananassae* reference RNA-seq virgin male testes
		Virgin female ovary	SRR2021003	42,009,242	*D. ananassae* reference RNA-seq virgin ovary
D. melanogaster	r6.17 (N=13,933)	Virgin male testis	SRR2021001	42,748,857	*D. melanogaster* RNA-seq virgin male testes
		Virgin female ovary	SRR2020999	44,104,658	*D. melanogaster* RNA-seq virgin female ovaries
D. simulans	r2.02 (N=14,179)	Virgin male testis	SRR1520537	64,659,682	*D. simulans* w501 reference RNA-seq virgin testes
		Virgin female ovary	SRR1511609	49,778,276	*D. simulans* w501 reference RNA-seq virgin ovary
D. yakuba	r1.05 (N=14,824)	Virgin male testis	SRR1693748	58,400,341	*D. yakuba* reference RNA-seq virgin testes
		Virgin female ovary	SRR1693746	60,228,477	D. *yakuba* reference RNA-seq virgin ovary

[a] The RNA-seq data listed were used for expression analyses with respect to tissue type, noting that when more than one RNA-seq dataset was available, the largest sample was used for study for parallel data sets in all contrasts. See Methods and Text File S5 for more details on RNA-seq datasets and analyses.

Table S2. The 17 tissues and developmental stages used to assess the breadth of expression, or pleiotropy, of sex-biased gene sets. Data was collected from modENCODE in FlyBase (flybase.org). Expression was defined as those with ≥1 RPKM as defined in the database.

Tissues and Stages Under Study
Imaginal disc, larvae L3 wandering
Nervous system larvae pupae
Head virgin 1,4,10 day female
Head mated 1,4,10 day female
Head mated 1,4,10 day male
Salivary gland larvae prepupae
Digestive system L3 day 1, 4 20 adult
Fat body l3 prepupae pupae p8
Carcass L3
Ovary mated 4 day female
Testis mated 4 day male
Embryo 2-24 hours
Larvae stage 1, 2,3
Larvae puff stage
Prepupae and pupae
Adult 1,5 and 20 day male
Adult 1,5 and 20 day female

Table S3. The clade-wide testis-specific genes (N=171) and their gene identifiers for *D. melanogaster* (www.flybase.org).

Fbgn ID	Gene Name	Fbgn ID	Gene Name	Fbgn ID	Gene Name
fbgn0025115	*Acyp*	fbgn0261806	CG42752	fbgn0034104	CG15705
fbgn0033952	*Adgf*	fbgn0261358	CG42635	fbgn0039810	CG15549
fbgn0035585	*ATPsynCF6L*	fbgn0259917	CG42446	fbgn0031130	CG15452
fbgn0031367	*c-cup*	fbgn0259729	CG42383	fbgn0034554	CG15227
fbgn0038714	*Cpr92A*	fbgn0037827	CG4073	fbgn0032709	CG15167
fbgn0029501	*Crtp*	fbgn0035069	CG3611	fbgn0034463	CG15125
fbgn0062411	*Ctr1C*	fbgn0031430	CG3528	fbgn0040694	CG14974
fbgn0038089	*d-cup*	fbgn0085331	CG34302	fbgn0032365	CG14929
fbgn0036808	*Dic4*	fbgn0085318	CG34289	fbgn0038218	CG14841
fbgn0036438	*goddard*	fbgn0085315	CG34286	fbgn0033278	CG14759
fbgn0034658	*Grx1t*	fbgn0085274	CG34245	fbgn0037987	CG14739
fbgn0031905	*gudu*	fbgn0085239	CG34210	fbgn0037986	CG14736
fbgn0036706	*ND-24L-PA*	fbgn0085206	CG34177	fbgn0037829	CG14691
fbgn0050073	*Obp50b*	fbgn0085204	CG34175	fbgn0037320	CG14668
fbgn0034468	*Obp56a*	fbgn0085199	CG34170	fbgn0034278	CG14488
fbgn0034129	*Parp16*	fbgn0054049	CG34049	fbgn0037191	CG14448
fbgn0035004	*Pgam5*	fbgn0054029	CG34029	fbgn0038097	CG14384
fbgn0017556	*Prosalpha4T2*	fbgn0053233	CG33233	fbgn0038124	CG14380
fbgn0051742	*Prosbeta5R2*	fbgn0052987	CG32987	fbgn0038158	CG14370
fbgn0028570	*robl22E*	fbgn0052986	CG32986	fbgn0038512	CG14330
fbgn0028944	*Semp1*	fbgn0052983	CG32983	fbgn0036089	CG14151
fbgn0037462	*sunz*	fbgn0052833	CG32833	fbgn0040814	CG14113
fbgn0050417	*tbrd*	fbgn0052718	CG32718	fbgn0260459	CG14106
fbgn0052463	*Tengl2*	fbgn0052651	CG32651	fbgn0029964	CG1409
fbgn0038458	*VhaM9.7*	fbgn0052488	CG32488	fbgn0032312	CG14071
fbgn0250827	*whip*	fbgn0052487	CG32487	fbgn0032315	CG14069
fbgn0033770	*wuc*	fbgn0052240	CG32240	fbgn0031721	CG14017
fbgn0051860	ZnT33D	fbgn0034825	CG3215	fbgn0039041	CG13838
fbgn0031444	CG9879	fbgn0047338	CG32148	fbgn0036708	CG13725

fbgn0039784	CG9698	fbgn0051933	CG31933	fbgn0265266	CG13639
fbgn0030624	CG9106	fbgn0051816	CG31816	fbgn0034869	CG13558
fbgn0033322	CG8584	fbgn0051730	CG31730	fbgn0034867	CG13557
fbgn0038127	CG8476	fbgn0051609	CG31609	fbgn0034841	CG13541
fbgn0036159	CG7557	fbgn0051515	CG31515	fbgn0034820	CG13538
fbgn0036161	CG7551	fbgn0051358	CG31358	fbgn0034774	CG13526
fbgn0032650	CG7094	fbgn0063261	CG31275	fbgn0033701	CG13171
fbgn0040989	CG5693	fbgn0051050	CG31050	fbgn0032188	CG13137
fbgn0035943	CG5653	fbgn0051008	CG31008	fbgn0032111	CG13110
fbgn0038386	CG5478	fbgn0050369	CG30369	fbgn0033141	CG12831
fbgn0038944	CG5388	fbgn0061435	CG30270	fbgn0038356	CG12784
fbgn0028884	CG4892	fbgn0050177	CG30177	fbgn0031388	CG12674
fbgn0039568	CG4815	fbgn0025838	CG2652	fbgn0032626	CG12620
fbgn0039029	CG4704	fbgn0037455	CG2336	fbgn0040871	CG12479
fbgn0267689	CG46025	fbgn0035384	CG2113	fbgn0032094	CG12439
fbgn0264543	CG43922	fbgn0039463	CG18472	fbgn0038558	CG12347
fbgn0264301	CG43779	fbgn0028856	CG18063	fbgn0032370	CG12307
fbgn0264081	CG43750	fbgn0034202	CG17287	fbgn0033280	CG12126
fbgn0263982	CG43731	fbgn0031410	CG17237	fbgn0036156	CG11726
fbgn0263403	CG43449	fbgn0036962	CG17122	fbgn0037572	CG11698
fbgn0263389	CG43441	fbgn0033828	CG17048	fbgn0036221	CG11588
fbgn0262961	CG43272	fbgn0035584	CG17030	fbgn0034713	CG11291
fbgn0262845	CG43209	fbgn0032109	CG17005	fbgn0029963	CG10920
fbgn0262812	CG43183	fbgn0035009	CG16837	fbgn0028858	CG10839
fbgn0262786	CG43167	fbgn0032503	CG16825	fbgn0032769	CG10750
fbgn0262592	CG43127	fbgn0042198	CG16741	fbgn0036327	CG10748
fbgn0262361	CG43059	fbgn0034505	CG16739	fbgn0260455	CG10332
fbgn0262144	CG42870	fbgn0030440	CG15719	fbgn0039083	CG10177

Table S4. The raw counts of genes with lineage-specific transitions (LSTs) in sex-biased expression status in the gonads.

Transition Type	No. of Lineage-specific transitions			
	Dmel	Dsim	Dyak	Dana
Testis-Biased				
Ov-Ts	4	5	8	31
Unb-Ts	152	187	296	206
Ovary-Biased				
Ts-Ov	51	34	11	46
Unb-Ov	160	116	140	276
Unbiased				
Ts-Unb	253	136	121	296
Ov-Unb	119	185	356	242

Table S5. The gene ontology (GO) functions of genes exhibiting lineage-specific transitions (LSTs) from testis to ovary-biased expression and those exhibiting LSTs from ovary-to testis-biased expression. For each transition type, genes were pooled across all species (due to the low number per species). The clustering by function was conducted in DAVID [2] using Drosophila gene identifiers, and the four clusters with the greatest enrichment score are shown per category. *P*-values are from a modified Fisher's test, wherein lower values indicate greater enrichment.

Ovary- to Testis Biased Transitions

**Cluster 1 Enrichment Score: 0.69**	P-value
Serine/threonine-protein kinase, active site	1.10E-01
Protein kinase, ATP binding site	1.30E-01
ATP binding	1.60E-01
S_TKc	1.90E-01
Protein kinase, catalytic domain	2.10E-01
protein phosphorylation	2.20E-01
Protein kinase-like domain	3.10E-01
Transferase	5.00E-01
**Cluster 2 Enrichment Score: 0.57**	
ATP binding	1.60E-01
ATP-binding	2.90E-01
Nucleotide-binding	4.20E-01
**Cluster 3 Enrichment Score: 0.54**	
Zinc finger C2H2-type/integrase DNA-binding domain	2.10E-01
Zinc finger, C2H2-like	2.80E-01
Zinc finger, C2H2	3.10E-01
ZnF_C2H2	3.80E-01
**Cluster 4 Enrichment Score: 0.07**	

Testis- to Ovary-Biased Transitions

**Cluster 1 Enrichment Score: 2.25**	P-value
Fatty acid biosynthesis	4.10E-03
Lipid biosynthesis	5.50E-03
Lipid metabolism	6.00E-03
Fatty acid metabolism	7.10E-03
**Cluster 2 Enrichment Score: 1.24**	
Oxidoreductase	4.20E-02
iron ion binding	4.50E-02
oxidation-reduction process	1.10E-01
**Cluster 3 Enrichment Score: 1.23**	
DNA-binding region: Homeobox	2.10E-05
Homeodomain	6.00E-04
HOX	6.40E-04
Homeobox	6.50E-04
Homeobox, conserved site	2.50E-03
Homeodomain-like	4.10E-03
brain development	5.00E-03
sequence-specific DNA binding	4.00E-02
transcription factor activity, sequence-specific DNA binding	4.80E-02
transcription factor activity, RNA polymerase II distal enhancer sequence-specific binding	9.40E-02

Transmembrane helix	8.20E-01	compositionally biased region: Poly-Ala	1.70E-01
Transmembrane	8.20E-01	regulation of transcription from RNA polymerase II promoter	2.10E-01
Membrane	8.90E-01	DNA-binding	3.00E-01
integral component of membrane	8.90E-01	positive regulation of transcription from RNA polymerase II promoter	3.50E-01
		Developmental protein	3.90E-01
		multicellular organism development	4.20E-01
		regulation of transcription, DNA-templated	5.10E-01
		dendrite morphogenesis	5.10E-01
		transcription, DNA-templated	6.80E-01
		Transcription	8.30E-01
		Transcription regulation	9.30E-01
		Nucleus	9.50E-01
		DNA binding	9.70E-01
		Nucleus	1.00E+00
		**Cluster 4 Enrichment Score: 1.21**	
		Leucine-rich repeat, typical subtype	4.50E-02
		LRR_TYP	4.80E-02
		Leucine-rich repeat	1.10E-01

Table S6. The gene ontology (GO) functions of genes exhibiting lineage-specific transitions (LSTs) from unbiased to testis- biased (unb-ts) expression and from unbiased to ovary-biased (unb-ov) expression for each of the four species under study. The clustering by function was conducted in DAVID (Huang da et al. 2009) using Drosophila gene identifiers. Functions were grouped by percentage of genes in each category in DAVID using the Chart option. Categories representing ≥2% of the gene set are shown. Olfactory functions are in bold italics. The number of genes per category is provided in Table S4.

**Unbiased to Testis-Biased Transitions**		**Unbiased to Ovary-Biased Transitions**	
**GO Term**	Percent	**GO Term**	Percent
**<u>Dmel</u>**		**<u>Dmel</u>**	
Signal	34.90	cytoplasm	18.1
Membrane	34.20	Coiled coil	16.9
Transmembrane helix	30.90	Transferase	14.4
Transmembrane	30.90	Alternative splicing	9.4
integral component of membrane	27.60	Cytoplasm	8.8
Receptor	12.50	mitochondrion	7.5
Disulfide bond	11.20	imaginal disc-derived wing morphogenesis	5
integral component of plasma membrane	8.60	Immunoglobulin-like fold	3.8
plasma membrane	8.60	identical protein binding	2.5
Transducer	7.20	Rab GTPase binding	2.5
Cell membrane	7.20	ubiquitin protein ligase activity	2.5
G-protein coupled receptor signaling pathway	5.30	protein ubiquitination involved in ubiquitin-dependent protein catabolic process	2.5
topological domain: Extracellular	5.30		
topological domain: Cytoplasmic	5.30		
Peptidase S1, trypsin family, active site	4.60		
Peptidase S1A, chymotrypsin-type	4.60		
Peptidase S1	4.60		
Trypsin-like cysteine/serine peptidase domain	4.60		
serine-type endopeptidase activity	4.60		
Tryp_SPc	4.60		
Neuroactive ligand-receptor interaction	3.90		
neuropeptide signaling pathway	3.90		
G-protein coupled receptor	3.90		
calcium ion binding	3.90		
SM01381	3.30		

ligand-gated ion channel activity	3.30		
G protein-coupled receptor, rhodopsin-like	3.30		
GPCR, rhodopsin-like, 7TM	3.30		
odorant binding	3.30		
olfactory receptor activity	3.30		
Serine protease	3.30		
neuropeptide receptor activity	2.60		
sensory perception of sound	2.60		
cell junction	2.60		
detection of chemical stimulus involved in sensory perception of smell	2.60		
Synapse	2.60		
microtubule-based movement	2.60		
male courtship behavior	2.60		
Cell junction	2.60		
synaptic transmission, cholinergic	2.00		
ovarian follicle cell stalk formation	2.00		
Neurotransmitter-gated ion-channel, conserved site	2.00		
Neurotransmitter-gated ion-channel transmembrane domain	2.00		
Neurotransmitter-gated ion-channel	2.00		
Neurotransmitter-gated ion-channel ligand-binding	2.00		
Low-density lipoprotein (LDL) receptor class A, conserved site	2.00		
neuropeptide hormone activity	2.00		
Low-density lipoprotein (LDL) receptor class A repeat	2.00		
LDLa	2.00		
detection of chemical stimulus	2.00		
**Dsim**		**Dsim**	
Signal	33	Metabolic pathways	16.7
Membrane	28.6	mitochondrion	11.4
Transmembrane helix	27	Oxidoreductase	11.4
Transmembrane	27	Transport	7.9
integral component of membrane	26.5	Oxidative phosphorylation	7
Disulfide bond	11.4	Mitochondrion	7
Receptor	8.1	lipid particle	7
sensory perception of pain	7	lateral inhibition	6.1
Cell membrane	6.5	response to oxidative stress	4.4

integral component of plasma membrane	6.5	determination of adult lifespan	4.4
proteolysis	5.9	carbohydrate metabolic process	3.5
Transducer	5.4	Armadillo-like helical	3.5
topological domain:Cytoplasmic	5.4	Lyase	3.5
topological domain: Extracellular	4.9	hydrogen-exporting ATPase activity, phosphorylative mechanism	2.6
disulfide bond	4.3	proton-transporting ATP synthase activity, rotational mechanism	2.6
olfactory receptor activity	3.8	ATP synthesis coupled proton transport	2.6
endomembrane system	3.8	proton transport	2.6
sweet taste receptor activity	3.2	Hydrogen ion transport	2.6
taste receptor activity	3.2	mitochondrial large ribosomal subunit	2.6
sensory perception of taste	3.2	wound healing	2.6
Ubl conjugation pathway	3.2		
sleep	3.2		
multicellular organism development	3.2		
response to carbon dioxide	2.7		
sensory perception of bitter taste	2.7		
chemosensory behavior	2.7		
7TM chemoreceptor	2.7		
male courtship behavior	2.7		
odorant binding	2.7		
dendrite	2.7		
axon	2.7		
neuronal cell body	2.7		
Ion channel	2.7		
circadian rhythm	2.2		
Protein processing in endoplasmic reticulum	2.2		
**Dyak**		**Dyak**	
Membrane	37.2	nucleus	24.3
Signal	35.1	cytoplasm	18.6
Transmembrane helix	34.8	Nucleus	15.7
Transmembrane	34.8	Transferase	12.1
integral component of membrane	33.8	neurogenesis	8.6
Oxidoreductase	11.1	Nucleotide-binding, alpha-beta plait	5.7
Metabolic pathways	10.8	nucleotide binding	5.7
Disulfide bond	8.8	RNA binding	5.7

Receptor	8.4	Spliceosome	5
plasma membrane	8.1	mRNA binding	4.3
Transport	7.4	Armadillo-type fold	4.3
sensory perception of pain	6.4	mRNA splicing, via spliceosome	4.3
oxidation-reduction process	5.7	mRNA processing	3.6
integral component of plasma membrane	5.7	regulation of alternative mRNA splicing, via spliceosome	3.6
Glycoprotein	5.7	Protein biosynthesis	3.6
transmembrane region	5.7	centrosome	3.6
membrane	5.1	Armadillo-like helical	3.6
glycosylation site: N-linked (GlcNAc...)	4.7	Serine/threonine-protein kinase, active site	3.6
Cell membrane	4.7	synaptic vesicle endocytosis	2.9
topological domain:Cytoplasmic	4.4	domain: RRM	2.9
Transducer	4.1	Transcription	2.9
topological domain: Extracellular	4.1	neuromuscular synaptic transmission	2.9
disulfide bond	3.7	poly(A) RNA binding	2.9
Calcium	3.4	protein folding	2.9
Biosynthesis of antibiotics	3.4	RNA processing and modification	2.1
Ion transport	3.4	positive regulation of Wnt signaling pathway	2.1
calcium ion binding	3.4	transcription elongation from RNA polymerase II promoter	2.1
Sensory transduction	3	protein import into nucleus	2.1
oxidoreductase activity	3	ubiquitin ligase complex	2.1
Carbon metabolism	2.7	positive regulation of ERK1 and ERK2 cascade	2.1
axon	2.7	compositionally biased region: Poly-Thr	2.1
chitin-based cuticle development	2.7	mRNA splicing	2.1
iron ion binding	2.7	positive regulation of Ras protein signal transduction	2.1
Immunoglobulin-like fold	2.7	spliceosomal complex	2.1
ligand-gated ion channel activity	2.4	Basal transcription factors	2.1
LRR_TYP	2.4		
Leucine-rich repeat, typical subtype	2.4		
Mitochondrion inner membrane	2.4		
Lysosome	2.4		
Glycosidase	2.4		
Leucine-rich repeat	2.4		
olfactory receptor activity	2.4		
Ion channel	2.4		

G-protein coupled receptor signaling pathway	2.4		
Oxidative phosphorylation	2.4		
detection of chemical stimulus	2		
phototransduction	2		
visual perception	2		
carbohydrate metabolic process	2		
Insect cuticle protein	2		
structural constituent of cuticle	2		
**Dana**		**Dana**	
Signal	30.7	nucleus	18.2
extracellular region	7.3	Coiled coil	17.1
signal peptide	6.8	Transferase	11.3
Receptor	6.8	Cytoplasm	10.9
transmembrane region	6.8	cytosol	9.8
Glycoprotein	6.3	Phosphoprotein	9.8
integral component of plasma membrane	5.9	neurogenesis	8
Cell membrane	5.4	Nucleotide-binding	7.3
glycosylation site: N-linked (GlcNAc...)	5.4	P-loop containing nucleoside triphosphate hydrolase	6.5
Immunoglobulin-like domain	4.9	WD40/YVTN repeat-like-containing domain	4.7
Immunoglobulin-like fold	4.9	WD40-repeat-containing domain	4.4
sequence-specific DNA binding	4.4	Golgi apparatus	4
neuronal cell body	3.9	WD40	4
Transducer	3.9	WD40 repeat	4
olfactory receptor activity	3.4	Pleckstrin homology-like domain	3.3
cilium assembly	2.9	GTP binding	3.3
Ion channel	2.9	dorsal closure	2.9
Sensory transduction	2.9	Activator	2.9
chitin-based cuticle development	2.9	Winged helix-turn-helix DNA-binding domain	2.9
sodium channel activity	2.4	protein homodimerization activity	2.9
Sodium channel	2.4	regulation of transcription from RNA polymerase II promoter	2.9
Na+ channel, amiloride-sensitive	2.4	WD repeat	2.5
cilium morphogenesis	2.4	sleep	2.5
Sodium transport	2.4	GTPase activity	2.5
Sodium	2.4	Hippo signaling pathway - fly	2.2
sodium ion transport	2.4	poly(A) RNA binding	2.2

detection of chemical stimulus involved in sensory perception of smell	2.4	ovarian follicle cell development	2.2
HOX	2.4	WD40 repeat, conserved site	2.2
IGc2	2.4	actin binding	2.2
IG	2.4	Golgi membrane	2.2
Homeobox, conserved site	2.4		
male courtship behavior	2.4		
structural constituent of chitin-based larval cuticle	2.4		
Homeobox	2.4		
odorant binding	2.4		
structural constituent of cuticle	2.4		
Homeodomain	2.4		
Insect cuticle protein	2.4		
Immunoglobulin subtype 2	2.4		
Immunoglobulin subtype	2.4		
heterophilic cell-cell adhesion via plasma membrane cell adhesion molecules	2		
cilium	2		
Hippo signaling pathway - fly	2		
sperm individualization	2		
dendrite membrane	2		
EGF-like, conserved site	2		
Immunoglobulin I-set	2		

Table S7. The genes with known olfactory functions that exhibited LSTs from unbiased to testis-biased status. Gene identifiers are for *D. melanogaster*. The species with the LST is shown.

Gene Identifier	Protein Name	Species with unb-ts Transition
fbgn0033508	Odorant-binding protein 46a(Obp46a)	Dmel
fbgn0033614	Odorant-binding protein 47b(Obp47b)	Dmel
fbgn0034473	Odorant receptor 56a(Or56a)	Dmel
fbgn0037685	Odorant receptor 85f(Or85f)	Dmel
fbgn0038203	Odorant receptor 88a(Or88a)	Dmel
fbgn0010403	Odorant-binding protein 83b(Obp83b)	Dsim
fbgn0011281	Odorant-binding protein 83a(Obp83a)	Dsim
fbgn0011281	Odorant-binding protein 83a(Obp83a)	Dsim
fbgn0026384	Odorant receptor 59a(Or59a)	Dsim
fbgn0033043	Odorant receptor 42b(Or42b)	Dsim
fbgn0036681	Odorant-binding protein 73a(Obp73a)	Dsim
fbgn0026399	Odorant receptor 85e(Or85e)	Dyak
fbgn0030298	Odorant receptor 10a(Or10a)	Dyak
fbgn0037399	Odorant receptor 83c(Or83c)	Dyak
fbgn0028946	Odorant receptor 35a(Or35a)	Dana
fbgn0030715	Odorant receptor 13a(Or13a)	Dana
fbgn0034475	Odorant-binding protein 56h(Obp56h)	Dana
fbgn0034766	Odorant-binding protein 59a(Obp59a)	Dana
fbgn0041622	Odorant receptor 69a(Or69a)	Dana

Table S8. The number of genes per fold-bias category for testis-biased and ovary-biased genes for all Drosophila species studied herein.

	Dmel	Dsim	Dyak	Dana
**Ovary-Biased**				
2-5 Fold	2334	2074	1823	2165
5-10 Fold	790	801	779	897
>10 Fold	447	446	509	527
Total	3571	3321	3111	3589
**Testis-Biased**				
2-5 Fold	838	806	819	795
5-10 Fold	513	446	427	394
>10 Fold	1968	2267	2637	2169
Total	3319	3519	3883	3358

Table S9. The gene ontology (GO) functions of genes with clade-wide testis-specific expression (N=171). The clustering by function was conducted in DAVID (Huang da et al. 2009) using Drosophila gene identifiers, and the four clusters with the greatest enrichment score are shown per category. *P*-values are from a modified Fisher's test, wherein lower values indicate greater enrichment.

**Cluster 1: Enrichment Score: 2.72**	P-value
ATPase activity, coupled	2.90E-04
dynein complex	1.70E-03
microtubule-based movement	1.40E-02
**Cluster 2: Enrichment Score: 2.14**	
phosphoprotein phosphatase activity	3.70E-04
HAD-superfamily hydrolase, subfamily IIA	1.90E-03
Nitrophenylphosphatase-like domain	1.90E-03
monoester phosphate phosphatase, PGP type	2.30E-03
protein dephosphorylation	2.90E-03
HAD-like domain	8.10E-03
phosphatase activity	1.40E-02
dephosphorylation	5.20E-02
Cytosol	9.80E-01
**Cluster 3: Enrichment Score: 1.65**	
integral component of membrane	1.40E-04
Transmembrane helix	9.40E-02
Transmembrane	9.60E-02
Membrane	2.00E-01
**Cluster 4: Enrichment Score: 1.46**	
EF-hand-like domain	1.10E-02
EF-Hand 1, calcium-binding site	2.40E-02
EF-hand domain	3.30E-02
calcium ion binding	1.80E-01

Table S10. The percentage of genes with clade-wide sex-biased status (SBS) exhibiting positive selection using sites analysis (M7 versus M8) in PAML [3] across all four species (Dmel, Dsim, Dyak, Dana). In this assessment, only genes with dN and dS<1.5 and dS>0.001 in all four species branches were used for analysis, with nearly all excluded genes resulting from the most divergent species Dana (note that in all other dN/dS analyses, only those in the branch of interest matching these criteria were excluded, making this assessment conservative, see Methods). M7 versus M8 results are also shown for sites analysis using the flyDIVaS database, which includes two additional species *D. sechellia* and *D, erecta* [4]. Different letters following percentage positive selection in a single row indicate a statistically significant difference using a Chi^2 test (P<0.05).

**Sites Analysis**	**Universally Testis-biased**	**Universally Ovary-biased**	**Universally Unbiased**
**Present Study (conservative)**			
N Total	2071	1966	1372
N Suitable range all species including Dana	683	827	813
N Positive Selection	85	84	77
Percent Positive Selection	12.45a	10.16a	9.47a
**Flydivas**			
N Total	2071	1966	1372
N Suitable range all species including Dana	1558	1554	1067
N Positive Selection	249	211	115
Percent Positive Selection	15.98a	13.58a	10.78b

Table S11. The percentage of genes with lineage-specific transitions (LSTs) in sex-biased status that exhibited positive selection in the target branch (with a LST) using branch-site analysis in PAML (P<0.05) [3]. For each gene studied, the species branch with the LST was defined as the target branch of interest for testing positive selection in branch-site analysis. The number of LSTs studied per species branch are provided in Table S4. Only genes studied with dN and dS<1.5 and dS>0.001 were included, and thus these are conservative estimates.

	**Dmel**	**Dsim**	**Dyak**	**Dana**
Testis-Biased				
Unb-Ts	5.37	12.22	11.94	27.20
Ovary-Biased				
Unb-Ov	5.63	14.68	14.62	25.45

Text File S1: Additional methods

Whilst the outgroup Dana was divergent to the ingroup clade containing Dmel, Dsim, and Dyak (Fig. S1), we found the majority of genes in that branch had dS <3 (9,370) and $dN<1.5$ (N=10,708), a suitable range for protein divergence methods, and thus the taxon was included in the determination of the four species dN/dS values. Nonetheless, under a conservative approach, for analysis of dN/dS in each species terminal branch including Dana we used only genes with values of dN and $dS<1.5$ and $dS>0.001$ for analysis. The N values for genes matching these stringent criteria (of 10,740 orthologous gene sets) wherein dN/dS was studied in the respective terminal branch were 10,557, 10,440, 10,001, and 5,340 for Dmel, Dsim, Dyak and Dana respectively. Thus, the gene set studied for Dana is smaller than for the three ingroup species, which include the vast majority of genes under study (N=10,740). Nonetheless, this represents a large gene sample size sufficient for study of dN/dS in Dana, and thus this terminal branch was included in our study of protein divergence.

Text File S2: Fold Bias

For testis-biased genes, the largest percentage belonged to the ≥10 fold-biased class (>59% of each testis-biased gene set), whereas the majority of ovary-biased genes were contained within the ≥2-5 fold category for each of the four species. This indicates that testis-biased gene expression is skewed toward higher fold-bias than ovary-biased expression (Table S8; Chi^2-tests of the ≥2-5 fold and of the ≥10-fold bias classes between testis- and ovary-biased genes, $P<0.0001$ per species).

As shown in Fig. S2, we found that an increase in fold testis-biased expression was associated with elevated expression in the testis, with the highest expression observed in the ≥10-fold class for each of four species (Ranked ANOVA and Dunn's paired contrast $P<0.05$ Fig. S2A-D). These high expression levels in the testis were accompanied by lower ovarian expression ($P<0.05$). For ovary-biased genes, the ≥10-fold class exhibited the highest levels of ovarian expression in each of the four Drosophila species ($P<0.05$, Fig. S2 E-H). Ovary-biased genes in the ≥10-fold class of expression also showed decreased testis expression as compared to the ≥2- to 5-fold, ≥5- to 10-fold classes. Thus, elevated levels of ovary-biased expression in these Drosophila species results from both upregulation in the ovaries and downregulation in the testes.

Our findings concur with those of our previous study on *A. aegypti*, wherein fold-bias in gonad expression was correlated with changes in transcript levels in both sexes [5]. Further interspecies gonadal data from a wider range of insect genera will help to determine the generality of these patterns.

Text File S3: Functions of genes with conserved testis-specific expression in all four species

Genes with conserved testis-specific expression across all four species are of particular interest as their conserved specificity implies that there has been a long-term selective advantage of exclusive expression in the male gonad. We thus conducted to GO analysis to assess the function of the universally testis-specific genes (N=171) and found that genes with the highest enrichment scores were are involved ATPase activity and membrane functions (Table S9). This is consistent with findings from mice showing that ATPase activity affects male fertility by regulating normal spermatogenesis and apoptosis [6], and from *D. melanogaster* showing that testis-biased genes contain an overrepresentation of genes involved in ATP biosynthesis [7].

Text File S4: Minimal differences in positive selection between testis- and ovary-biased genes

To further evaluate the role of pleiotropy on dN/dS, particularly with respect to the other feasible hypothesis of adaptive evolution, we assessed positive selection for the universally testis-biased, ovary-biased and unbiased genes using maximum likelihood codon “sites” analyses in PAML [3]. For this, we compared the models M7 versus M8. The former model permits negative selection and neutral evolution, and the latter additionally allows for positive selection at codon sites [3]. As positive selection is highly sensitive to alignments and divergence level, we studied only the subset of genes with dN and dS <1.5 and dS>0.001 in all of the four species, including in the divergent species Dana, making this analysis highly conservative. As shown in Table S10, using genes matching these criteria, we found signatures of positive selection in 12.4%, 10.2% and 9.5% of genes that were universally testis-biased, ovary biased and unbiased respectively ($P<0.05$ for 2ΔlnL). These signatures were consistent with mildly, but not statistically significantly, higher frequency of genes with positive selection in the testis-biased gene set (Chi^2 $P>0.05$ for paired contrasts).

For additional rigor, we examined positive selection tests for the melanogaster group available from flyDIVaS [4, 8]. This database includes two additional species in addition to the four examined here (*D. sechellia* and *D. erecta*), and should be an indicator of the proportion of genes evolving adaptively in this clade. Using that database (and genes with successful orthology calls and appropriately unsaturated alignments therein), we found signatures of positive selection in 16.0, 13.6, and 10.8% of the universally testis-biased, ovary-biased and unbiased genes respectively. For each of these categories, the proportion of genes with such signatures was higher than our calculated estimates based on our four study species, consistent with inclusion of more species. Nonetheless, whilst the group of universally testis-biased genes had more instances of positive selection than the comparable ovary-biased genes, only its comparison to unbiased genes was statistically significant (Chi^{-2} $P<0.05$). Further, the actual difference between testis-biased and ovary-biased genes was again small (net difference in percentages was <2.5%). Together, these data suggest that while positive selection is more common overall in testis-biased than ovary-biased genes, this difference only affects a small subset of genes (difference ≤2.5% using either approach). Extensive positive selection has been thought to be characteristic of male-sexual genes for Drosophila [9-11], but this has not always been found in this taxon [12], nor in other organisms [5]. Our results suggest a weak effect for the universally testis-biased genes examined here.

In order to test for adaptive evolution accompanying transitions to sex-biased expression, we examined those genes exhibiting LSTs from unbiased to sex-biased status, that is unb-ts and unb-ov (Table 1), using branch-site analyses [3], where the species branch containing the LST was chosen as the foreground (tested) branch for each gene [3]. We report that branch-site positive selection was observed for both types of transitions in each species, with a low of 5.4% for genes with unb-ts LSTs in Dmel up to 27.2% for unb-ts LSTs in the comparatively much longer branch Dana. However, there were minimal differences in the proportion of unb-ts and unb-ov genes exhibiting positive selection within each species terminal branch. Specifically, the net differences observed between unb-ts and unb-ov were less than 2.7% between the two LST categories for each species, with unb-ov having mildly higher values than unb-ts for Dmel, Dsim and Dyak and the opposite trend detected for Dana. This analysis thus indicates no evidence of greater instances of positive selection for LSTs to testis-biased expression than to ovary-biased expression (Table S11). In sum, a mildly elevated level of positive selection was observed for universally testis-biased genes, and no effect for LSTs, suggesting that the strong and persistent rapid evolution of testis-biased genes observed here (Figs. 5, S3) is better explained by low pleiotropy (Fig. 6) than by adaptive sequence evolution.

Text File S5: The current approach and RNA-seq data

The RNA-seq data in Table S1 used for the present study comprise deep sequence datasets, with >42 million paired-end reads per sample/tissue, which were utilized to generate FPKM per gonadal tissue per species. Use of deep large-scale and single RNA-seq samples (per tissue) has been repeatedly employed to comparatively study tissue expression in metazoans [13-16], including Drosophila studies based on public data such as Drosophila modENCODE [15, 17] and sex-biased gene expression [1, 14]; the latter is facilitated by very high correlations observed among replicates of sex-related samples (see below, e.g., [18]). In a similar manner, single expressed-sequence tags (ESTs) datasets (per tissue) have been repeatedly and effectively used to characterize and compare expression within and among tissues, including sexual tissues [7, 19-24].

In our assessment, we determined testis and ovary expression levels using mapping of the deep RNA-seq samples (Table S1) to species-specific CDS using Geneious 11.0.3 [25] as outlined in Methods. Very strong correlations were found across all 10,770 genes in ovary FPKM (Spearman's $R=0.93$, $P<2X10^{-7}$) and in testis FPKM ($R=0.87$, $P<2X10^{-7}$) between the two most closely related species Dmel and Dsim (Fig. 2B) affirming the effectiveness of these large-scale read samples for precisely quantifying sex-related expression profiles (Fig. 2B) (see also below).

While Dmel and Dana RNA-seq had unitary samples in the available gonadal data sets [1], Dsim and Dyak had two or three replicates (that were provided at the SRA); for comparability of sampling per species, we used the single or the largest RNA-seq dataset that was available per taxon for study (Table S1). For additional stringency, we wished to assess FPKM for a species using replicated data for comparison to our results obtained using data from Table S1. For this assessment, we downloaded the second largest ovary and testis RNA-seq sets for Dsim, as LSTs in that branch occurred most recently (5 My, than Dyak). Identifiers for ovary and testis replicates used for Dsim at the SRA database are SRR1511608, SRR1548741; the testes RNA-seq sample had 14 million fewer paired-reads than replicate 1 (Table S1) while ovaries had similar read counts (less than 300,000 fewer reads than in Table S1).

We found FPKM values using the two RNA-seq data sets for the ovaries and for the testes were each very strongly correlated in Dsim. Specifically, the Spearman's ranked R for FPKM between replicates across all 10,740 genes was 0.96 for the ovaries and was 0.94 for testes, $P<2X10^{-7}$. Furthermore, the ratio of testis:ovary expression, measured as $\log_2$ (testis FPKM/ovary FPKM), across all genes was also strongly correlated $R=0.86$, $P<2X10^{-7}$ between the two replicates. Thus, FPKM

values in testes and in ovaries were remarkably consistent between the two replicates in Dsim, as was the degree of gonadal sex-biased expression (fold testis:ovary bias), affirming very high reproducibility of each of the single sex-related gene sets.

We next conducted differential expression analyses using P-values from Deseq2 [26] and the two replicates (average values per tissue, FPKM ≥1 in one tissue type) for testes and for ovaries in Dsim (denoted as Approach 2), and compared the findings to our main results in our study (denoted as Approach 1; Fig. 1). We found that the vast majority of genes, 88.9%, had the same categorical SBS using the Approach 1 in our main study (Table S1) and using Approach 2 of the replicated datasets. Most of the variation, 8.9%, between the two approaches was, as expected, from genes near the threshold of sex-biased (2 to 5 fold) and unbiased status. No genes, that is 0%, had opposite (sex-bias) calls between testis-biased and ovary-biased status between the two methods (Table S1). Thus, using replicated samples and Deseq2 resulted in a good agreement with the main analyses; nonetheless, the former more conservative approach tended to classify more genes near the cutoff of sex-biased expression as unbiased.

Finally, we compared our classifications of LSTs using data in Table S1 to that obtained using the replicated testis-ovary datasets. Using the more conservative Approach 2, we found that the majority of Dsim genes with LSTs (75.1%) retained the same categorical LST status as found in our main study (Approach 1; Table S4). However, a subset of genes exhibited their ancestral state under Approach 2 (values of 0, 0, 3.7, and 17.3% of the observed LSTs for ov-ts, ts-ov ts-unb, ov-unb respectively), particularly for unb-ov and unb-ts (48.3 and 22.4% per transition type). This is consistent with more conservative calls (under Approach 2) near the sex-biased expression threshold, and particularly for unb-ov as those LSTs had had lower fold-bias than unb-ts (see Fig. 4; note that sex-bias status changes are sensitive to methods and cutoffs, the latter of which are in-effect arbitrary [27]; thus the same approach should be employed for all contrasts, as conducted in our main study). Excluding all genes with any variation in SBS between the two approaches, that is using the largest RNA-seq dataset in Table S1 and using replicated (averaged expression) and Deseq2 analyses, still yielded a more than three-fold higher level of ts-ov than ov-ts (one-sided Chi^2 P=0.025) and a markedly lower rate of reversals in SBS than gains/losses (at least 6-fold for each type of contrast, P<0.0001). Thus, both approaches yielded similar patterns as observed in Table 2 and Table S4, indicating the results are robust to the method employed.

Using results from the RNA-seq data in our study (Table S1), we found strong correlations in gonad (for testis and for ovary) expression (FKPM) across all genes per sex between Dmel and Dsim

(Fig. 2B), and between replicates studied here, concurring with precise depiction of expression profiles. Further, a stepwise decrease in correlations in expression level (FPKM) was observed with time (Fig. 1), which was particularly marked for testis-expression, consistent with Drosophila whole male-female analyses [27]. In addition, all 171 genes identified as clade-wide testis-specific (Table S3) using the datasets here were 100% confirmed using data from Dmel at the modENCODE (http://www.modencode.org) database [15]. Collectively, these patterns affirm stringency of the unitary datasets in Table S1 for the study of sex-biased expression evolution in these taxa.

Supplementary References

1. Rogers RL, Shao L, Sanjak JS, Andolfatto P, Thornton KR: **Revised annotations, sex-biased expression, and lineage-specific genes in the Drosophila melanogaster group**. *G3 (Bethesda)* 2014, **4**(12):2345-2351.
2. Huang da W, Sherman BT, Lempicki RA: **Systematic and integrative analysis of large gene lists using DAVID bioinformatics resources**. *Nat Protoc* 2009, **4**(1):44-57.
3. Yang Z: **PAML 4: phylogenetic analysis by maximum likelihood**. *Molecular Biology and Evolution* 2007, **24**(8):1586-1591.
4. Stanley CE, Jr., Kulathinal RJ: **flyDIVaS: A Comparative Genomics Resource for Drosophila Divergence and Selection**. *G3 (Bethesda)* 2016, **6**(8):2355-2363.
5. Whittle CA, Extavour CG: **Rapid Evolution of Ovarian-Biased Genes in the Yellow Fever Mosquito (Aedes aegypti)**. *Genetics* 2017, **206**(4):2119-2137.
6. Jaiswal MK, Agrawal V, Katara GK, Pamarthy S, Kulshrestha A, Chaouat G, Gilman-Sachs A, Beaman KD: **Male fertility and apoptosis in normal spermatogenesis are regulated by vacuolar-ATPase isoform a2**. *Journal of Reproductive Immunology* 2015, **112**:38-45.
7. Meisel RP: **Towards a more nuanced understanding of the relationship between sex-biased gene expression and rates of protein-coding sequence evolution**. *Mol Biol Evol* 2011, **28**(6):1893-1900.
8. Clark AG, Eisen MB, Smith DR, Bergman CM, Oliver B, Markow TA, Kaufman TC, Kellis M, Gelbart W, Iyer VN *et al*: **Evolution of genes and genomes on the *Drosophila* phylogeny**. *Nature* 2007, **450**(7167):203-218.
9. Swanson WJ, Clark AG, Waldrip-Dail HM, Wolfner MF, Aquadro CF: **Evolutionary EST analysis identifies rapidly evolving male reproductive proteins in Drosophila**. *Proceedings of the National Academy of Sciences of the United States of America* 2001, **98**:7375-7379.
10. Zhang Z, Hambuch TM, Parsch J: **Molecular evolution of sex-biased genes in Drosophila**. *Mol Biol Evol* 2004, **21**(11):2130-2139.
11. Haerty W, Jagadeeshan S, Kulathinal RJ, Wong A, Ram KR, Sirot LK, Levesque L, Artieri CG, Wolfner MF, Civetta A *et al*: **Evolution in the fast lane: rapidly evolving sex-related genes in Drosophila**. *Genetics* 2007, **177**:1321-1335.
12. Dorus S, Busby SA, Gerike U, Shabanowitz J, Hunt DF, Karr TL: **Genomic and functional evolution of the *Drosophila melanogaster* sperm proteome**. *Nature Genetics* 2006, **38**(12):1440-1445.

13. Brawand D, Soumillon M, Necsulea A, Julien P, Csárdi G, Harrigan P, Weier M, Liechti A, Aximu-Petri A, Kircher M *et al*: **The evolution of gene expression levels in mammalian organs**. *Nature* 2011, **478**:343-348.
14. Assis R, Zhou Q, Bachtrog D: **Sex-biased transcriptome evolution in Drosophila**. *Genome Biol Evol* 2012, **4**(11):1189-1200.
15. Graveley BR, Brooks AN, Carlson JW, Duff MO, Landolin JM, Yang L, Artieri CG, van Baren MJ, Boley N, Booth BW *et al*: **The developmental transcriptome of *Drosophila melanogaster***. *Nature* 2011, **471**(7339):473-479.
16. Kaiser VB, Zhou Q, Bachtrog D: **Nonrandom gene loss from the Drosophila miranda neo-Y chromosome**. *Genome Biol Evol* 2011, **3**:1329-1337.
17. Li JJ, Huang H, Bickel PJ, Brenner SE: **Comparison of *D. melanogaster* and *C. elegans* developmental stages, tissues, and cells by modENCODE RNA-seq data**. *Genome research* 2014, **24**(7):1086-1101.
18. Congrains C, Campanini EB, Torres FR, Rezende VB, Nakamura AM, Oliveira JLd, Lima ALA, Chahad-Ehlers S, Sobrinho IS, Brito RAd: **Evidence of Adaptive Evolution and Relaxed Constraints in Sex-Biased Genes of South American and West Indies Fruit Flies (Diptera: Tephritidae)**. *Genome Biology and Evolution* 2018, **10**(1):380-395.
19. Duret L, Mouchiroud D: **Expression pattern and, surprisingly, gene length shape codon usage in Caenorhabditis, Drosophila, and Arabidopsis**. *Proc Natl Acad Sci U S A* 1999, **96**(8):4482-4487.
20. Ewing RM, Ben Kahla A, Poirot O, Lopez F, Audic S, Claverie JM: **Large-scale statistical analyses of rice ESTs reveal correlated patterns of gene expression**. *Genome Res* 1999, **9**(10):950-959.
21. Duret L, Mouchiroud D: **Determinants of substitution rates in mammalian genes: expression pattern affects selection intensity but not mutation rate**. *Mol Biol Evol* 2000, **17**(1):68-74.
22. Park SG, Choi SS: **Expression breadth and expression abundance behave differently in correlations with evolutionary rates**. *BMC Evol Biol* 2010, **10**:241.
23. Parisi M, Nuttall R, Naiman D, Bouffard G, Malley J, Andrews J, Eastman S, Oliver B: **Paucity of genes on the Drosophila X chromosome showing male-biased expression**. *Science* 2003, **299**:697-700.
24. Mank JE, Hultin-Rosenberg L, Zwahlen M, Ellegren H: **Pleiotropic constraint hampers the resolution of sexual antagonism in vertebrate gene expression**. *American Naturalist* 2008, **171**:35-43.
25. Kearse M, Moir R, Wilson A, Stones-Havas S, Cheung M, Sturrock S, Buxton S, Cooper A, Markowitz S, Duran C *et al*: **Genious Basic: an integrated and extendable desktop software platform for the organization and analyiss of sequence data**. *Bioinformatics* 2012, **28**(12):1647-1649.
26. Love MI, Huber W, Anders S: **Moderated estimation of fold change and dispersion for RNA-seq data with DESeq2**. *Genome Biol* 2014, **15**(12):550.
27. Zhang Y, Sturgill D, Parisi M, Kumar S, Oliver B: **Constraint and turnover in sex-biased gene expression in the genus Drosophila**. *Nature* 2007, **450**(7167):233-237.
